# Nutritional risk stratification management is associated with reduced disease relapse and improved quality of life in IBD patients: a retrospective study

**DOI:** 10.3389/fnut.2025.1729247

**Published:** 2026-01-12

**Authors:** Xia Li, Shuli Ma, Xuxin Zhang, Hongmei He

**Affiliations:** Department of Nursing, Affiliated Hospital of Nantong University, Nantong, China

**Keywords:** disease relapse, inflammation control, inflammatory bowel disease, nutritional risk stratification, quality of life, retrospective study

## Abstract

**Objectives:**

To investigate the effects of nutritional risk stratification management on relapse control and quality of life (QoL) in patients with Inflammatory Bowel Disease (IBD), and to evaluate the clinical value of systematic nutritional intervention in the comprehensive management of IBD.

**Methods:**

This single-center retrospective cohort study analyzed 752 IBD patients (408 with ulcerative colitis and 344 with Crohn’s disease) who were managed in the Department of Gastroenterology at the Affiliated Hospital of Nantong University between January 2024 and December 2024. Patients were classified into an intervention group if they were managed under an institutional nutritional risk stratification pathway, which included dietitian-led screening with NRS-2002 or MUST and individualized nutrition plans with scheduled reassessment. Patients who received conventional care without entering this structured pathway served as the comparison group. All data, including nutritional status, inflammatory markers, disease activity, relapse events, hospitalization outcomes, and quality of life (IBDQ), were obtained retrospectively from electronic medical records and follow-up databases. Time-to-relapse was analyzed with Cox regression, and changes in continuous outcomes were evaluated using linear mixed-effects models. Propensity score matching and inverse probability of treatment weighting were used to mitigate measured confounding.

**Results:**

In this observational analysis, significant improvements from baseline were observed in the intervention group for body weight, BMI, and laboratory nutritional indicators (all *p* < 0.001). Increases in albumin and prealbumin showed a significant negative correlation with the decrease in inflammatory markers (*r* = −0.42, *p* < 0.001). The median CRP level decreased from 15.2 to 9.6 mg/L (*p* < 0.001), and fecal calprotectin decreased from 298 to 184 μg/g (*p* < 0.001). Disease activity scores (HBI and Mayo) significantly decreased in both CD and UC patients (all *p* < 0.001). After a median follow-up of 9.8 months, the relapse rate was significantly lower in the intervention group (20.5%) compared to the control group (34.8%) (*p* < 0.001). Cox regression analysis indicated that nutritional management was independently associated with a reduced risk of relapse (adjusted HR = 0.61, 95% CI: 0.46–0.83, *p* = 0.001). Furthermore, the intervention group had significantly lower hospitalization rates (12.7% vs. 20.9%, *p* = 0.009) and shorter hospital stays (6 days vs. 8 days, *p* = 0.015). Regarding QoL, the total IBDQ score increased significantly in the intervention group (Δ = +13.5, *p* < 0.001), and the significant time × group interaction (*p* < 0.001) suggested sustained, cumulative improvement. Subgroup and sensitivity analyses yielded consistent results, supporting the robustness of this association (IPTW HR = 0.59, 95% CI: 0.44–0.81, *p* = 0.001).

**Conclusion:**

In this single-center retrospective cohort, exposure to nutritional risk stratification management was associated with better nutritional status, lower inflammatory markers and disease activity, reduced relapse and hospitalization rates, and improved quality of life in IBD patients. These findings support the hypothesis that integrating structured nutritional risk screening and individualized nutrition management into routine IBD care may be beneficial. However, given the observational design and potential residual confounding, the observed associations should not be interpreted as definitive causal effects, and prospective multicenter randomized studies are needed to confirm these results and guide clinical practice.

## 1 Introduction

Inflammatory Bowel Disease (IBD), comprising Crohn’s disease and ulcerative colitis, is a chronic, relapsing condition with a rising global prevalence and substantial healthcare burden (1–5). A pivotal contributor to its poor prognosis is malnutrition, which arises from a detrimental cycle of reduced intake, malabsorption due to inflamed mucosa, and inflammation-driven hypermetabolism (6–10). The prevalence of malnutrition is high and variable (20%–85%), often accompanied by specific micronutrient deficiencies, and is strongly linked to infections, poor wound healing, and worsened outcomes (8–12).

Crucially, malnutrition is not merely a consequence but also a driver of disease activity. Impaired nutritional status significantly elevates the risk of relapse and is independently associated with reduced quality of life in IBD patients (13, 14). This underscores why nutritional assessment is a cornerstone of management. International guidelines accordingly recommend routine nutritional risk screening using validated tools like NRS-2002 and MUST to enable early identification and intervention (15, 16).

Nutritional intervention has become an integral component of comprehensive IBD therapy. Studies have shown that oral nutritional supplement (ONS) in Crohn’s disease patients in remission can significantly prolong the duration of remission (17). For patients with active disease, exclusive enteral nutrition (EEN) has been demonstrated to induce remission. In adult CD patients, EN is effective in inducing clinical remission (albeit slightly less so than corticosteroids), while in pediatric CD it is regarded as a first-line therapy (18). According to ESPEN guidelines, enteral nutrition should be prioritized in IBD patients with inadequate oral intake due to inflammation or complications, to maintain intestinal integrity and improve disease outcomes (19). Parenteral nutrition (PN) is indicated when EN is not feasible or when severe intestinal dysfunction exists (20). These international studies and guidelines underscore the importance of establishing nutritional support strategies; however, further work is needed to clarify how best to implement structured, risk-stratified nutritional care pathways in routine IBD management.

Although nutritional screening and support therapy are recognized as crucial aspects of IBD management, there is still no universally adopted, standardized protocol for how best to operationalize nutritional risk–stratified care in routine practice. Existing studies, including nutrition-focused interventions, screening-based approaches, and partial enteral nutrition programmes, have provided important insights, but real-world evaluations of structured, dietitian-led nutritional pathways remain relatively limited. This study adds real-world evidence from a large single-center cohort implementing a structured nutritional risk-stratification pathway. We classified and managed IBD patients according to their nutritional risk level and evaluated the association between this pathway and relapse rates, inflammatory markers, hospitalization outcomes, and quality of life. The aim is to assess whether nutrition support guided by routine nutritional risk assessment is associated with improved IBD prognosis, and to provide incremental data that may inform future development of IBD nutrition management strategies and support clinical decision-making. In this context, our retrospective cohort analysis should be viewed as a pragmatic contribution to the growing body of evidence on integrating nutritional risk management into comprehensive IBD care, rather than as establishing a conceptually novel intervention model.

## 2 Materials and methods

### 2.1 Study design and participants

This single-center, retrospective cohort study enrolled patients with IBD who were hospitalized or received outpatient follow-up in three departments of the Gastroenterology Division at Affiliated Hospital of Nantong University between 1 January 2024 and 31 December 2024. According to hospital statistics, the total number of IBD patients across these three departments during this period was approximately 1,892. After systematic screening, patients lacking complete medical records, nutritional screening records, or with a follow-up duration of less than 3 months were excluded. A total of 752 patients meeting the study criteria were ultimately included [408 with Ulcerative Colitis (UC) and 344 with Crohn’s Disease (CD)], representing approximately 38% of the annual case load. This sample size was deemed sufficient for the statistical analysis of the primary outcomes. The dietitian-led nutritional risk stratification pathway had been implemented at our center as a routine quality-improvement program before the study period. During routine care in 2024, NRS-2002 or MUST screening was performed either as part of this structured pathway (dietitian-led, with scheduled re-assessments) or as isolated opportunistic assessments by physicians or nurses. The present study used retrospectively collected clinical data without modifying any diagnostic or therapeutic procedures.

For each eligible patient, an index date (time zero) was defined as the date of the first IBD-related inpatient admission or outpatient visit at our institution in 2024 at which all inclusion criteria were met and an NRS-2002 or MUST screening had been completed or was scheduled within the subsequent 7 days. All baseline covariates, including nutritional status, disease activity and medication use, were defined with reference to this index date. Follow-up for relapse and other outcomes started at the index date and continued until the first relapse event, loss to follow-up, death, or 31 December 2024, whichever occurred first.

As the research involved the analysis of pre-existing, anonymized data from routine clinical care without any additional intervention or risk to patients, the Ethics Committee granted a waiver of the requirement for written informed consent.

**FIGURE 1:**
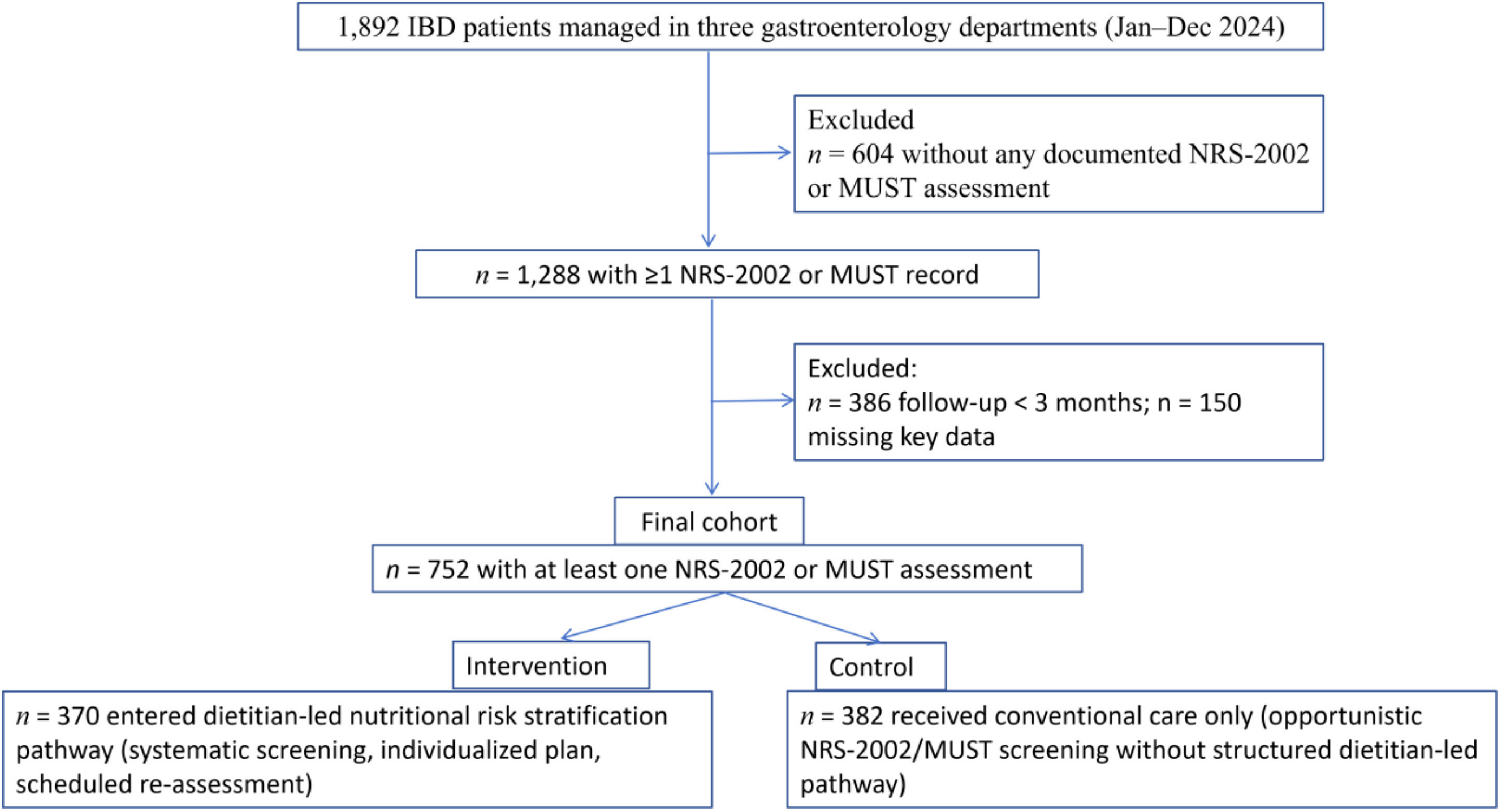
STROBE-compliant flow diagram of patient selection. Among 1,892 inflammatory bowel disease (IBD) patients managed in three gastroenterology departments between January and December 2024, patients were sequentially excluded for the following reasons: no documented NRS-2002/MUST nutritional screening (*n* = 604), follow-up <3 months (*n* = 386), and missing key clinical or outcome data (*n* = 150). The final analytic cohort comprised 752 patients with at least one NRS-2002/MUST record and ≥3 months of follow-up, who were further classified into the dietitian-led nutritional risk stratification management group and the conventional-care control group according to actual care pathways. Numbers at each stage are shown separately for patients potentially eligible for the nutritional pathway and those receiving only conventional care.

Patient selection is summarized in a STROBE-compliant flow diagram (Figure 1).

### 2.2 Inclusion and exclusion criteria

#### 2.2.1 Inclusion criteria

1. Age ≥ 18 years;
2. Diagnosis of UC or CD confirmed according to national IBD diagnostic criteria, based on endoscopic, histological, and/or radiological evidence;
3. Completion of at least one nutritional risk screening (NRS-2002 or MUST) at the hospital during the study period (1 January 2024–31 December 2024), irrespective of whether the assessment was completed by a registered dietitian, physician, or nurse;
4. Availability of complete clinical data, laboratory results, and follow-up records, with a minimum follow-up duration of 3 months;
5. Traceable baseline disease assessment and treatment information, with clearly documented disease type and activity level (as per electronic medical records or diagnostic reports);
6. Reliable sources for clinical data and follow-up information, with independent verification and consensus by two investigators.

#### 2.2.2 Exclusion criteria

Patients meeting any of the following criteria were excluded:

1. Comorbid conditions including malignancy, active tuberculosis, severe infection, severe hepatic or renal insufficiency, or pregnancy/lactation;
2. Missing key data (e.g., nutritional risk score, relapse records, or laboratory parameters) or loss to follow-up;
3. Receipt of PN or specific dietary interventions [e.g., EEN, Crohn’s Disease Exclusion Diet (CDED)] within the 3 months prior to the study baseline;
4. Presence of other comorbidities significantly affecting nutritional status or quality of life (e.g., severe heart failure, advanced liver disease, chronic pancreatitis);
5. Inconsistent or erroneous medical record information that could not be confirmed upon review.

Notably, completion of at least one NRS-2002 or MUST assessment during the study period was required for eligibility. In some patients, this assessment consisted of a single opportunistic screening performed by physicians or nurses during routine care and was not embedded within a structured dietitian-led management pathway. In others, NRS-2002 or MUST screening was performed by registered dietitians as part of the institutional nutritional risk stratification pathway, with predefined re-assessments. All available NRS-2002/MUST scores, regardless of the assessor, were extracted from the electronic medical record for the present analysis. Patients whose NRS-2002/MUST assessment was linked to a formal stratified nutrition pathway were classified into the intervention group, whereas patients with only opportunistic or physician/nurse-led screening and no structured follow-up were eligible for inclusion in the comparison (control) group.

### 2.3 Grouping and intervention modality

For the purposes of this retrospective analysis, exposure was defined according to the actual care pathway documented in the electronic medical records. Patients were assigned to the intervention group if they had at least one dietitian-led nutritional risk screening (NRS-2002 or MUST) with a documented individualized nutrition plan and at least one follow-up reassessment under the institutional nutrition pathway. By design, all included patients—both in the intervention and control groups—had at least one documented NRS-2002 or MUST assessment; however, in the control group this assessment could be performed opportunistically by physicians or nurses during routine care and did not trigger a standardized dietitian-led pathway. All other eligible patients, who received conventional gastroenterology care without entering this structured pathway, were classified into the control group.

Allocation to these groups was determined by routine clinical practice rather than randomization and may therefore be subject to confounding by indication. At our institution, referral into the dietitian-led pathway was typically initiated by the treating gastroenterologist or ward nurse when a positive NRS-2002/MUST screen and/or clinical features suggesting clinically relevant malnutrition (such as recent weight loss, poor oral intake, or active disease) were present, in the context of patient preference and dietitian availability. There was no single mandatory numerical threshold or fully standardized algorithm for referral during the study period, and the intensity and components of the pathway (for example, dietary education alone versus combinations with ONS, EN, or PN, and the frequency of reassessment) could vary between patients. Patients were therefore divided into two groups based on whether they received this dietitian-led nutritional risk stratification management pathway or conventional care alone.

For the primary analysis, exposure status (intervention vs. control) was assigned at baseline (time zero). Patients were classified into the intervention group if they entered the dietitian-led nutritional pathway at or within 14 days of the index date and had a documented individualized nutrition plan plus at least one scheduled follow-up visit with a registered dietitian. Patients who did not meet these criteria within this predefined baseline window were classified as controls. To avoid attributing “immortal” person-time to the intervention group, patients who were initially managed with conventional care but entered the structured pathway more than 14 days after the index date contributed person-time to the control group up to the date of crossover and were censored at that time in the primary Cox models. In a sensitivity analysis, entry into the nutritional pathway was additionally modeled as a time-varying covariate, with similar estimates for the association between nutritional management and relapse risk (see section “2.5 Statistical analysis” and Supplementary Table 1).

(1)    Intervention group

As part of the institutional nutrition pathway, patients in the intervention group underwent nutritional risk screening by a registered dietitian during admission or at the initial outpatient visit using the NRS-2002 or MUST tool (21). A positive screen was defined as an NRS-2002 score ≥ 3 or a MUST score ≥ 1. Based on the screening results, patients were stratified into three nutritional risk levels: Low Risk (NRS-2002 < 3 or MUST = 0), Moderate Risk (NRS-2002 = 3 or MUST = 1), and High Risk (NRS-2002 ≥ 4 or MUST ≥ 2). Screen-positive patients underwent a comprehensive nutritional assessment, including dietary intake status, weight change, body mass index (BMI), and laboratory parameters [e.g., serum albumin (Alb), prealbumin (PAB), hemoglobin (Hb)], which informed the development of an individualized intervention plan. Nutritional interventions were delivered by specialist dietitians in collaboration with clinicians, adhering to the principles of “stratified management, individualized intervention, and dynamic assessment” to ensure scientific rigor and continuity of care. The following description reflects the standard protocol at our center; in routine practice, individual patients could deviate from this protocol according to clinical judgment and patient preference.

1. Dietary education and counseling: Low-risk patients received primarily dietary education, involving face-to-face counseling by a registered dietitian within 72 h of admission or at the first outpatient visit. Education covered energy and protein requirement assessment (recommended energy 25–30 kcal/kg/day, protein 1.2–1.5 g/kg/day), adjustment of food energy density, dietary diversity, dietary fiber and fluid intake, and avoidance of excessive fasting or repeated dietary restrictions. Personalized dietary advice tailored to individual taste preferences and symptom profiles was provided, supplemented with written materials or mobile educational tools (e.g., WeChat mini-program) to enhance adherence. Follow-up occurred every 4 weeks to monitor weight, food intake, and symptom changes.
2. ONS: Patients at moderate nutritional risk received ONS in addition to dietary education, providing an additional 200–400 kcal per day (equivalent to 1–2 standard bottles). High-protein formulas (≥4 g protein per 100 kcal) or anti-inflammatory formulas containing ω-3 fatty acids were recommended to support intestinal mucosal repair. In cases of mild lactose intolerance, low-lactose or peptide-based formulas were substituted. ONS was advised to be taken in 2–3 divided doses between meals to improve absorption and prevent early satiety. Tolerance, proportion of energy intake from ONS, and intestinal responses were assessed every 2 weeks, with adjustments made to the formula and dosage accordingly.
3. EN: EN was initiated for high-risk patients or those whose oral intake was less than 60% of the recommended energy needs. Oral intake was prioritized where possible; otherwise, nasogastric or nasojejunal tubes were used. Formula selection criteria included: medium-chain triglyceride (MCT) content ≥ 40%, protein source comprising a whey-to-casein ratio of approximately 6:4; peptide-based or semi-elemental formulas were used in cases of concomitant malabsorption or active inflammation. Energy targets were set at 20–30 kcal/kg/day, protein at 1.2–1.5 g/kg/day, with non-protein energy distributed between carbohydrates and fat at a 55:45 ratio. Administration methods included intermittent bolus feeding (250–300 mL per session, every 3–4 h) or continuous infusion, starting at a rate of 80–100 mL/h and gradually reaching the target volume within 24–48 h. The EN course lasted 2–8 weeks, during which weight, intestinal tolerance, and adverse reactions (e.g., bloating, diarrhea) were assessed weekly, and the prescription was adjusted based on laboratory parameters (Alb, CRP, blood glucose).
4. PN: PN was reserved for patients with severe intestinal dysfunction or complete intolerance to EN (e.g., intestinal obstruction, short bowel syndrome, severe postoperative inflammation). Nutritional targets were 25–30 kcal/kg/day for energy and 1.3–1.5 g/kg/day for protein, with a glucose-to-fat energy ratio maintained at 6:4 and a nitrogen-to-non-protein energy ratio of 1:130–150. PN was administered via central venous access, with infusion rate and composition dynamically adjusted based on daily energy expenditure and electrolyte balance. To prevent complications, blood glucose, electrolytes, liver enzymes, renal function, and triglyceride levels were monitored every 48–72 h. Upon recovery of intestinal function, patients were transitioned stepwise to EN and subsequently to oral intake.
5. Dynamic re-evaluation and intervention adjustment: All patients in the intervention group underwent dynamic re-evaluation every 2–4 weeks. Re-assessment included changes in weight, BMI, laboratory parameters (Alb, PAB, CRP), dietary intake, bowel symptoms, and disease activity scores [Harvey-Bradshaw Index (HBI) or Mayo score]. Based on the evaluation results, the Medical Nutrition Therapy (MNT) team could adjust the intervention pathway, such as escalating from ONS to EN, or transitioning from EN to oral maintenance. Patients demonstrating improved nutritional status (Alb ≥ 40 g/L and stable weight for ≥3 weeks) were transferred to a maintenance management phase.
6. Intervention adherence and quality control: The intervention plan, implementation details, and follow-up results for each patient were reviewed and approved by both the dietitian and the attending physician. Adherence was defined as “good” if re-evaluation records existed and were consecutive for ≥2 sessions. Reasons for interrupted intervention or refused follow-up were documented and considered in sensitivity analyses. An internal quality control mechanism was established, involving quarterly random audits of 10% of cases by a senior dietitian to verify consistency between screening forms, intervention records, and follow-up data. All intervention procedures strictly followed the hospital’s nutritional management protocols to ensure data traceability and research reproducibility. To characterize the actual implementation of the pathway beyond the theoretical targets, we extracted dietitian documentation on prescribed and achieved energy and protein intake for each visit. For each patient, the mean daily energy intake (kcal/kg/day) and protein intake (g/kg/day) actually consumed during the intervention period were calculated and expressed as a proportion of the individual prescribed targets; these data are summarized as medians (interquartile ranges) in Supplementary Table 2. In addition, we recorded, for each patient, which nutritional modalities were received (dietary education only, education plus ONS, EN, PN, or combinations thereof), the timing and direction of any escalation (e.g., education → ONS → EN → PN), and the duration of each modality (weeks from initiation to step-down or discontinuation). Follow-up intensity was quantified as the number of in-person dietitian visits and structured telephone contacts per patient over 12 months, and adherence was operationalized as the proportion of planned dietitian contacts actually attended and, where available, the proportion of days on which ≥75% of the prescribed ONS or EN volume was documented as consumed. Descriptive distributions of these intervention components, durations, and adherence metrics for the intervention group are presented in Supplementary Table 2.

(2)    Control group

Patients in the control group were not enrolled in the systematic nutritional risk stratification management pathway. They received only conventional disease treatment and follow-up management. All patients were treated and monitored according to the hospital’s standard IBD diagnostic and therapeutic protocol. Management was conducted by the same gastroenterology physician team to ensure consistency in disease treatment strategies and follow-up schedules with the intervention group, thereby minimizing potential treatment confounding effects.

Conventional management included disease activity assessment, pharmacological therapy, management of complications, and general health education. Pharmacological regimens were tailored according to the patient’s disease type and activity level, primarily involving 5-aminosalicylic acid (5-ASA), corticosteroids, immunomodulators (such as azathioprine or methotrexate), and biologics (such as anti-TNF-α agents or anti-integrin antibodies), combined with small molecule targeted therapies (e.g., tofacitinib or upadacitinib) when necessary. Dosage and regimen adjustments were determined by the attending physician based on disease activity and patient tolerance. For patients developing complications such as intestinal strictures, bleeding, or perforation, the hospital’s unified IBD surgical and perioperative management protocol was followed.

All patients underwent routine vital signs monitoring and laboratory tests during hospitalization and outpatient follow-ups, including complete blood count, C-reactive protein (CRP), erythrocyte sedimentation rate (ESR), liver and renal function, blood glucose, electrolytes, and fecal calprotectin (FCP) to assess disease activity and inflammatory status. By study design, all patients in this group had at least one NRS-2002 or MUST score recorded during routine care (as required for study inclusion), but they were not enrolled in the structured, dietitian-led nutritional risk stratification pathway. Any nutritional risk information, when available, was used at the discretion of the treating physician and was not linked to a predefined algorithm of dietitian-delivered assessment, intervention, and scheduled follow-up. Physicians might inquire about dietary habits during routine consultations and offer general lifestyle advice, such as avoiding spicy, high-fat, or hard-to-digest foods and increasing fluid and dietary fiber intake. However, no specific energy or protein intake targets were set, and no formal written or electronic nutritional follow-up plan was initiated. If patients voluntarily consumed ONS or health formula powders based on personal choice or online information during the treatment period—without professional dietitian evaluation, prescription, or scheduled follow-up—this behavior was considered an unstructured individual action and not counted as part of the intervention. In data recording, such individuals were retained but separately flagged for subsequent sensitivity analysis. The follow-up cycle for control group patients was typically every 2–3 months, encompassing condition assessment, laboratory testing, medication adherence evaluation, and treatment regimen adjustments. All follow-ups were conducted by the same clinical team to maintain consistency in information collection and decision-making criteria. During follow-up, physicians could provide general advice regarding disease progression, medication side effects, or signs of malnutrition but did not initiate formal nutritional consultation procedures or intervention records. In the main analysis, these patients remained in the control group, but we additionally performed per-protocol sensitivity analyses in which such patients were excluded or, alternatively, reclassified as partially exposed; the results of these analyses were similar to those of the primary analysis (Supplementary Table 3).

Regarding data management, all clinical, laboratory, and follow-up data for the control group were extracted from the hospital’s electronic medical record system. Following data entry, two researchers independently reviewed the data; discrepancies were resolved by a third researcher to ensure data completeness and accuracy. For patients lost to follow-up or with missing data, the research team documented specific reasons (e.g., relocation, withdrawal from follow-up, or referral) and included them in the missing data analysis module for multiple imputation processing. Ethically, control group patients received the same rights to information and privacy protection management as the intervention group. All medical practices adhered to the hospital’s clinical routines, and the study did not intervene in or pose any risk to their treatment plans. The establishment of this group served as a conventional treatment control, aimed at comparing the clinical effects and disease outcomes of the systematic nutritional management model.

### 2.4 Outcome measures

#### 2.4.1 Nutritional risk and nutritional status indicators

For this analysis, baseline nutritional risk and nutritional status were defined using the first available NRS-2002 or MUST assessment during the study period and the corresponding anthropometric and laboratory data recorded in the electronic medical record. Nutritional risk was assessed using the NRS-2002 or the MUST. In the intervention group, these assessments were systematically performed by registered dietitians at admission or the initial outpatient visit, at the time of entry into the institutional nutrition pathway, and inpatients were re-screened within 7–10 days as part of structured follow-up, with all results documented in the electronic medical record (21). In the control group, the baseline NRS-2002 or MUST score was derived from routine clinical care and could be completed opportunistically by physicians or nurses according to hospital policy; these assessments were not linked to a predefined algorithm of dietitian-delivered assessment, intervention, and scheduled re-evaluation. An NRS-2002 score ≥ 3 or a MUST score ≥ 1 was defined as indicative of nutritional risk. All available NRS-2002/MUST scores, irrespective of the assessor, were extracted from the electronic medical record for the present study. Because NRS-2002 and MUST have different scoring structures and were developed in different settings, we did not pool their raw scores directly. Instead, each patient was classified into one of three harmonized nutritional risk categories (low, moderate, high) according to the thresholds recommended in the original validation studies (NRS-2002: 0–2, 3–4, ≥5; MUST: 0, 1, ≥2). These three-level categories were then used as indicators of baseline nutritional risk in the main analyses, irrespective of the specific tool applied. As a sensitivity analysis, key models were repeated in the subset of patients assessed with NRS-2002 only; the pattern and magnitude of associations were similar (Supplementary Table 4).

Anthropometric measurements included height, weight, body mass index (BMI = weight/height^2^), and percentage weight change [%ΔBW = (current weight–baseline weight)/baseline weight × 100%]. Weight was measured using the same electronic scale (calibration error ≤ 0.1 kg) each morning under fasting conditions while wearing light clothing. BMI was expressed in kg/m^2^. Weight loss ≥ 5% was defined as mild malnutrition, and ≥10% as significant malnutrition.

Laboratory nutritional indicators included serum Alb, PAB, Hb, and total lymphocyte count (TLC). All blood samples were collected in the morning under fasting conditions and analyzed by the hospital’s clinical laboratory center using automated biochemical analyzers. Alb < 35 g/L, PAB < 0.15 g/L, and TLC < 1.2 × 10°/L were defined as indicative of inadequate nutritional reserves. Inflammation-related markers included C-reactive protein (CRP), erythrocyte sedimentation rate (ESR), and fecal calprotectin (FCP). CRP was measured via immunoturbidimetry (mg/L), and FCP was determined using enzyme-linked immunosorbent assay (ELISA) (μg/g), serving as an important biomarker for intestinal inflammatory activity.

The aforementioned nutritional indicators were reassessed at baseline, mid-follow-up (approximately 3–6 months), and the final follow-up (12 months). All analyses were performed by the same laboratory to ensure longitudinal comparability.

#### 2.4.2 Disease activity and relapse indicators

Disease activity was assessed by attending gastroenterologists at enrollment and during each follow-up visit. Crohn’s disease patients were evaluated using the HBI (22), while ulcerative colitis patients were assessed with the Mayo score system (23). Active disease was defined as an HBI ≥ 8 or a Mayo score ≥ 6. Disease activity assessment integrated laboratory inflammatory markers (CRP, ESR, FCP) and imaging/endoscopic findings to reduce bias from any single indicator.

Relapse was defined as a significant exacerbation of disease activity during the study follow-up period. A relapse event was confirmed if any of the following criteria were met: (1) Requirement to re-initiate or intensify systemic corticosteroid therapy due to worsened condition; (2) FCP > 250 μg/g accompanied by an increase in symptom scores or deterioration in laboratory inflammatory markers; (3) Endoscopic or imaging evidence showing significant worsening in the extent or severity of inflammation compared to previous assessments; (4) Emergency department visit or hospitalization due to worsened IBD activity.

The time to relapse was defined as the date when any of the above criteria were first met. Time-to-event was calculated from the index date (time zero) defined in Section 2.1 to the date of the first relapse or censoring. To avoid information bias, the collection and adjudication of relapse data were performed blinded to patient group allocation. Relapse rate (%), time to relapse (days), and number of relapses were included in subsequent statistical analyses. Relapse events and follow-up information were ascertained from scheduled outpatient visits, inpatient records, and structured telephone follow-up documented in the hospital databases; nonetheless, relapses managed at other institutions may have been missed, which could lead to under-ascertainment of events.

Relapse events were identified from hospital databases and confirmed by two independent gastroenterologists according to the pre-defined criteria. Disagreements were resolved by consensus or by a third senior gastroenterologist.

#### 2.4.3 Quality of life indicators

Patient quality of life was assessed using the Inflammatory Bowel Disease Questionnaire-32 (IBDQ-32) (24). This scale comprises 32 items covering four domains: bowel symptoms, systemic symptoms, emotional function, and social function. Each item is scored from 1 to 7, yielding a total score ranging from 32 to 224, with higher scores indicating better quality of life. If patients completed only a short version of the IBDQ (IBDQ-9 or IBDQ-10), scores were converted to IBDQ-32 equivalent values using a standardized formula: (short-form total score÷number of items in the short form) × 32 (25).

The questionnaire was administered face-to-face by uniformly trained assessors in a quiet environment, with language assistance or explanations provided as necessary. Assessments were conducted at baseline enrollment and at the final follow-up (12 months). If a patient discontinued follow-up prematurely due to disease relapse, the last available IBDQ assessment was considered the endpoint data. The change in quality of life (ΔIBDQ = IBDQfinal–IBDQbaseline) served as one of the primary outcomes, evaluating the impact of nutritional stratification management on patients’ overall health perception and psychosocial function.

#### 2.4.4 Other outcome measures

In addition to the primary outcomes, several secondary indicators were collected to reflect disease control and clinical outcomes. Firstly, IBD-related hospitalizations during the study period were recorded, including annual hospitalization rate (%), mean number of hospitalizations, and length of stay per hospitalization (days), to assess the impact of nutritional management on healthcare resource utilization. Secondly, rescue medication use was documented, including the initiation or escalation of corticosteroids, biologics, or small molecule targeted agents (e.g., tofacitinib or upadacitinib), reflecting changes in treatment intensity. Thirdly, longitudinal changes in nutritional and laboratory indicators were monitored, including differences in weight, BMI, Alb, PAB, and Hb between baseline and the final follow-up, to quantify the degree of nutritional improvement.

Furthermore, any surgical interventions, serious adverse events (SAEs), or deaths occurring during the follow-up period were meticulously recorded and included in the safety analysis. The definitions and measurement methods for all outcome measures were pre-specified during the study protocol development phase and approved by the Ethics Committee. Following data entry, all data were independently verified by two researchers to ensure accuracy, completeness, and traceability.

### 2.5 Statistical analysis

All statistical analyses were performed using SPSS version 27.0 (IBM Corp., Armonk, NY, United States) and R software version 4.3.0 (R Foundation for Statistical Computing, Vienna, Austria). Prior to analysis, data completeness and normality were assessed. The distribution of continuous variables was determined using the Shapiro-Wilk test. Continuous data are presented as mean ± standard deviation (x̄ ± SD) or median (interquartile range, IQR) based on their distribution characteristics. Categorical variables are described using frequencies (*n*) and percentages (%). All tests were two-sided, with a statistical significance level set at *p* < 0.05.

Before database lock, we pre-specified the primary outcome (time to first relapse) and its main multivariable Cox model, as well as key secondary outcomes including relapse frequency, IBD-related hospitalization, change in IBDQ total score, and changes in serum albumin and CRP. Additional analyses, such as detailed subgroup comparisons beyond the pre-defined strata, E-value analysis, and exploratory models of other laboratory biomarkers, were considered exploratory and are presented as hypothesis-generating.

Baseline characteristics were compared using appropriate statistical methods to ensure intergroup balance. To evaluate the representativeness of the final cohort and the potential for selection bias, we conducted additional descriptive comparisons. First, we compared basic demographic and clinical characteristics (age, sex, disease type, markers of disease activity and inflammation, and department of care) between included and excluded patients. Second, we compared patients with at least one documented NRS-2002/MUST screening record and those without screening among the 1,892 IBD patients managed during the study period. Continuous variables were compared using *t*-test or Mann–Whitney U test, and categorical variables using χ^2^ test or Fisher’s exact test as appropriate. These comparisons are presented in Supplementary Tables 5, 6 to allow readers to judge the representativeness of the analytic cohort and to contextualize potential selection mechanisms. To further describe the complexity and heterogeneity of the nutritional intervention, we summarized the distribution of intervention components within the dietitian-led pathway. Specifically, we calculated the proportion of patients receiving dietary education only, education plus ONS, EN, PN, or combinations of these modalities; the median duration (weeks) of each modality; and the intensity of follow-up (number of dietitian contacts) in the intervention group. We also quantified the ratio of achieved to prescribed energy and protein intake for each patient. These descriptive data are reported in Supplementary Table 2 and are intended to help readers interpret the “dose” and composition of nutritional exposure. To address potential contamination in the control group, we pre-specified per-protocol sensitivity analyses excluding patients who self-initiated ONS or other substantial dietary changes without dietitian involvement, as well as an analysis reclassifying such patients as partially exposed; results are presented in Supplementary Table 3.

For continuous variables conforming to a normal distribution, independent samples *t*-tests were used; for variables deviating from normality, the Mann-Whitney U test was employed. Comparisons of categorical variables utilized the χ^2^ test or Fisher’s exact test, depending on sample size and expected frequencies. For variables with imbalanced sample sizes between groups or substantial missing data, weighted adjustments or sensitivity analyses were conducted to ensure the robustness of the findings.

The primary outcome was the time to first relapse event, with “receipt of nutritional risk stratification management” as the primary exposure variable. Time-to-event analysis for relapse was performed using Cox proportional hazards regression models to calculate hazard ratios (HR) and 95% confidence intervals (CI). The proportional hazards assumption was verified using Schoenfeld residual tests. Multivariable models were adjusted for potential confounders of relapse risk, including age, sex, disease type (UC or CD), disease duration, baseline disease activity score, serum Alb, CRP, BMI, and medication categories (5-ASA, corticosteroids, immunomodulators, biologics, or small molecule targeted agents). The inclusion of these covariates was based on previous literature evidence and clinical relevance. In addition, we constructed an *a priori* directed acyclic graph (DAG; Supplementary Figure 1) to represent our assumptions about the relationships between nutritional management, disease activity, comorbidities, health-care utilization, and relapse risk, and used this framework to guide the selection of covariates for the Cox models and propensity score analyses. Time-to-event analysis for relapse was performed using Cox proportional hazards regression models to calculate hazard ratios (HR) and 95% confidence intervals (CI). With 208 relapse events in the analytic cohort, the ratio of events to covariates in the primary Cox model exceeded 10 events per parameter, which reduces the risk of model overfitting.

In the primary Cox models, exposure to nutritional risk stratification management was treated as a fixed baseline covariate defined at time zero, based on whether the patient had entered the dietitian-led pathway within the prespecified 14-day window after the index date. Patients who crossed over from conventional care to the structured pathway after this window contributed follow-up time as unexposed (control) until the date of crossover and were censored at that time. To further address the possibility of immortal time bias, we performed an additional time-dependent Cox analysis in which entry into the nutritional pathway was modeled as a time-varying covariate that changed from 0 to 1 on the date of first dietitian-led pathway entry; the estimated association between nutritional management and relapse risk remained materially unchanged (Supplementary Table 1).

Analyses of secondary outcomes included relapse frequency, changes in quality of life, hospitalization rates, and rescue medication use. Relapse frequency, being count data, was analyzed using Negative Binomial Regression to estimate intergroup differences, adjusting for potential overdispersion. Changes in quality of life (IBDQ total score) between baseline and the final follow-up were analyzed using a Linear Mixed-Effects Model (LMM), incorporating a “time × group” interaction term to assess the impact of nutritional risk stratification management on the trajectory of quality of life over time. This model included random effects for individuals to account for within-subject correlation due to repeated measures. Hospitalization rates and rescue medication use (including treatment escalation with corticosteroids, biologics, or small molecule drugs), as binary variables, were analyzed using Logistic Regression models to calculate odds ratios (OR) and 95% CIs, adjusted for potential confounders.

To reduce selection bias and confounding effects, the study further employed Propensity Score Matching (PSM) and Inverse Probability of Treatment Weighting (IPTW) for balance correction. The propensity score was estimated using a logistic regression model with “receipt of stratified management” as the dependent variable and covariates including demographic characteristics, disease features, and nutritional indicators. PSM was performed using 1:1 nearest neighbor matching with a caliper width of 0.2. Balance between groups after matching was assessed using the Standardized Mean Difference (SMD), with SMD < 0.1 considered indicative of good balance. To validate the robustness of the matching results, IPTW was also used for re-weighting analysis, calculating the Average Treatment Effect (ATE) in the weighted pseudo-population and comparing it with the original results.

Standardized mean differences for all covariates before and after matching/weighting are displayed in Supplementary Figure 2, and the effective sample size of the IPTW pseudo-population is reported in Supplementary Table 7.

Furthermore, several sensitivity analyses were conducted to test model robustness. Firstly, the primary analysis was repeated using a stricter and more objective relapse definition that required FCP > 250 μg/g together with either IBD-related hospitalization or endoscopic/imaging evidence of worsening inflammation, in order to verify the robustness of the main findings to a biomarker- and event-based outcome definition. Detailed results of this objective-definition sensitivity analysis are presented in Supplementary Table 8.

For missing data in key covariates, Multiple Imputation (*m* = 20) was employed. The imputation model included age, sex, BMI, Alb, CRP, disease type, activity score, and medication variables. Multiple Imputation by Chained Equations (MICE) was used to generate 20 imputed datasets. Statistical models were run independently on each dataset, and the results were pooled using Rubin’s rules. The pooled estimates were compared with the results from the original analysis to verify the robustness of the conclusions.

To control the risk of false positives due to multiple comparisons in the analysis of multiple outcomes, the Benjamini-Hochberg procedure for False Discovery Rate (FDR) correction was applied to all outcomes except the primary one. The FDR procedure was applied to the family of secondary outcomes, including relapse frequency, IBD-related hospitalization, rescue medication use, change in IBDQ, and changes in continuous nutritional and inflammatory markers. In Tables 2–5 we report raw (unadjusted) *p*-values, whereas FDR-adjusted *p*-values for the secondary outcomes are provided in an additional column in Supplementary Table 9. All statistical tests were two-sided with a significance level of *p* < 0.05. Results for continuous variables are reported as Mean Differences (MD) or regression coefficients (β), and results for categorical variables are reported as OR or HR. The interpretation of final results was based on both 95% confidence intervals and statistical significance, ensuring the scientific validity and reproducibility of the conclusions.

Given the non-randomized, retrospective nature of the study, all comparisons between the intervention and control groups should be interpreted as associations rather than causal effects. Multivariable adjustment, propensity score matching, and IPTW can mitigate confounding due to measured covariates, but they cannot fully account for unmeasured or imperfectly measured factors (e.g., disease severity, health-seeking behavior, or adherence). Therefore, residual confounding and selection bias remain possible despite these analytic strategies.

### 2.6 Ethics and data protection

This study strictly adhered to the Strengthening the Reporting of Observational Studies in Epidemiology (STROBE) guidelines. As this was a retrospective observational study based on pre-existing medical record information, involving no interventional procedures or additional examinations, the Ethics Committee granted a waiver for obtaining written informed consent from patients. The design and conduct of the study complied with the principles of the Declaration of Helsinki (2013 revision) and China’s “Measures for Ethical Review of Biomedical Research.” The dietitian-led nutritional risk stratification pathway evaluated in this study had been implemented as part of routine clinical care and institutional quality improvement, independent of the present research. The study team did not influence clinical decisions, the choice of nutritional management, or the follow-up schedule.

Regarding data handling, all research data were sourced from the hospital’s Electronic Medical Record (EMR) system and follow-up databases. Data extraction was performed by two researchers according to a standardized procedure after ethics approval. During extraction, all directly identifiable personal information, including names, national identification numbers, hospital admission numbers, and contact details, was removed. Data underwent de-identification and anonymization before import into the statistical analysis platform, where unique codes replaced identifiable information, ensuring that individuals could not be re-identified through backward tracing. All research databases were stored on secure hospital servers with multi-level access controls, restricting access only to authorized members of the research team.

Furthermore, to comply with data security and research integrity requirements, the study strictly followed relevant laws and regulations, including the “Personal Information Protection Law of the People’s Republic of China” and the “General Data Protection Regulation (GDPR, EU 2016/679).” All research materials were used solely for the purposes of this scientific investigation and not for any commercial or non-research purposes. The publication and reporting of study results present data only in aggregated statistical form, containing no individually identifiable information, thereby ensuring the full protection of patient privacy.

## 3 Results

### 3.1 Patient screening and baseline characteristics

Between January and December 2024, a total of 1,892 patients with IBD were managed across three departments of the Gastroenterology Division at Affiliated Hospital of Nantong University. Following the application of inclusion and exclusion criteria based on data completeness, 604 patients lacking nutritional risk screening records, 386 with follow-up duration of less than 3 months, and 150 with missing key data were excluded. Consequently, 752 eligible patients were ultimately enrolled (enrollment rate: 39.8%). This cohort included 408 patients (54.3%) with UC and 344 patients (45.7%) with CD. A detailed flow diagram of patient selection is shown in Figure 1. Supplementary Table 5 compares basic demographic and clinical characteristics between the 752 included patients and the 1,140 excluded patients. Supplementary Table 6 compares patients with and without documented NRS-2002/MUST screening among the full 1,892-patient source population. These descriptive analyses indicate that, while the distributions of age, sex, and disease type were broadly similar, there were some differences in markers of disease activity and health-care utilization between groups, highlighting the possibility of selection mechanisms related to disease severity, screening practices, and follow-up patterns. By design, all included patients had at least one documented NRS-2002 or MUST score in the electronic medical record, and the first available score during the study period was used as the baseline nutritional risk measure for both groups. Based on whether they received systematic nutritional risk stratification management, patients were categorized into an intervention group (*n* = 370, 49.2%) and a control group (*n* = 382, 50.8%).

None of the baseline characteristics assessed—including age, sex, BMI, proportion with significant weight loss, disease type, disease duration, smoking status, surgical history, anatomical distribution (CD), disease behavior phenotype (CD), disease activity scores, CRP, ESR, FCP, Alb, PAB, Hb, TLC, NRS-2002 score, and MUST score—showed statistically significant differences between the two groups prior to the intervention (all *p* > 0.05, Table 1). This indicates that the study groups were overall well-balanced at baseline, providing a solid statistical foundation and enhancing internal validity for the subsequent evaluation of intervention effects and multivariable model analyses.

**TABLE 1:** Baseline characteristics of patients.

Variable	Intervention group (*n* = 370)	Control group (*n* = 382)	*P*-value
Age (years, mean ± SD)	41.9 ± 13.1	41.3 ± 13.4	0.62
Sex (male, %)	52.2	53.7	0.74
BMI (kg/m^2^, mean ± SD)	21.4 ± 2.8	21.8 ± 3.0	0.28
Weight loss ≥ 5% in 3 months (%)	23.2	25.1	0.54
Disease type (UC/CD,%)	54.6/45.4	54.0/46.0	0.89
Disease duration (years, median [IQR])	5.1 [2.3–9.1]	5.4 [2.0–8.8]	0.48
Smoking (%)	21.6	22.8	0.73
Previous IBD surgery (%)	13.8	14.2	0.87
CD ileal involvement (%)	40.8	41.2	0.92
CD colonic involvement (%)	36.7	37.9	0.81
CD ileocolonic involvement (%)	22.5	20.9	0.66
CD behavior (B1/B2/B3, %)	57.9/26.8/15.3	59.2/27.2/13.6	0.69
HBI (CD, median [IQR])	7 [5–9]	6 [5–8]	0.31
Mayo score (UC, median [IQR])	6 [4–8]	5 [4–7]	0.42
CRP (mg/L, median [IQR])	13.5 [8.0–22.6]	12.9 [7.4–21.9]	0.58
ESR (mm/h, mean ± SD)	24.8 ± 13.2	23.6 ± 12.7	0.37
FCP (μg/g, median [IQR])	275 [190–380]	268 [185–365]	0.49
Albumin (g/L, mean ± SD)	38.6 ± 5.0	39.0 ± 5.2	0.44
Prealbumin (g/L, mean ± SD)	0.19 ± 0.06	0.20 ± 0.05	0.36
Hemoglobin (g/L, mean ± SD)	124.3 ± 15.7	125.8 ± 16.1	0.41
TLC (× 10^9^/L, mean ± SD)	1.64 ± 0.42	1.67 ± 0.45	0.52
NRS-2002 score (mean ± SD)	2.2 ± 1.1	2.1 ± 1.0	0.39
MUST score (mean ± SD)	0.7 ± 0.6	0.6 ± 0.6	0.41
5-ASA use (%)	61.3	60.7	0.86
Corticosteroid use (%)	27.1	26.7	0.91
Immunomodulator use (%)	18.4	17.8	0.82
Biologic/small-molecule use (%)	20.3	21.5	0.74
Annual hospitalizations [median (IQR)]	1 [0–2]	1 [0–2]	0.68

UC, ulcerative colitis; CD, Crohn’s disease; CRP, C-reactive protein; ESR, erythrocyte sedimentation rate; FCP, fecal calprotectin; TLC, total lymphocyte count; 5-ASA, 5-aminosalicylic acid.

### 3.2 Nutritional risk stratification and changes in nutritional status

Patients in the intervention group underwent nutritional risk screening at baseline enrollment and were categorized into three classes based on NRS-2002 or MUST scores: low risk (*n* = 102, 27.6%), moderate risk (*n* = 158, 42.7%), and high risk (*n* = 110, 29.7%). Baseline NRS-2002 or MUST scores were also available for control-group patients (Table 1), but, by design, these scores were obtained during routine care and did not automatically trigger enrollment into the structured, dietitian-led nutritional pathway. The distribution of intervention components (education, ONS, EN, PN, and their combinations), their median duration, and follow-up intensity within the intervention group is summarized in Supplementary Table 2.

Patients in the intervention group were stratified by baseline nutritional risk, with moderate and high-risk categories together accounting for approximately 70% of the cohort, underscoring the high prevalence of nutritional vulnerability in this IBD population (Table 2). As anticipated, baseline albumin, prealbumin, and body weight exhibited a graded decrease across ascending risk levels (*p* for trend <0.001).

**TABLE 2:** Changes in nutritional indicators within and between groups.

Variable	Intervention baseline	Intervention follow-up	Δ (within group, *p*)	Control baseline	Control follow-up	Δ (within group, *p*)	Between-group Δ (*p*)
Body weight (kg, mean ± SD)	—	+1.8 ± 3.4	+1.8 (*p* < 0.001)	—	−0.2 ± 2.1	−0.2 (p = 0.41)	<0.001
BMI (kg/m^2^, mean ± SD)	21.1 ± 2.8	22.0 ± 2.9	+0.9 (*p* < 0.001)	21.8 ± 3.0	21.7 ± 3.0	−0.1 (*p* = 0.39)	<0.001
Albumin (g/L, mean ± SD)	37.4 ± 5.2	40.1 ± 4.6	+2.7 (*p* < 0.001)	40.3 ± 4.9	40.5 ± 5.0	+0.2 (p = 0.55)	<0.001
Prealbumin (g/L, mean ± SD)	0.18 ± 0.05	0.22 ± 0.06	+0.04 (*p* < 0.001)	0.20 ± 0.06	0.20 ± 0.06	0 (*p* = 0.78)	<0.001
Hemoglobin (g/L, mean ± SD)	115.3 ± 16.7	121.9 ± 15.9	+6.6 (*p* = 0.004)	124.8 ± 16.1	125.0 ± 15.8	+0.2 (*p* = 0.71)	0.003
NRS-2002 (score, mean ± SD)	2.6 ± 1.1	1.9 ± 0.9	−0.7 (*p* < 0.001)	1.9 ± 1.0	1.8 ± 0.9	−0.1 (*p* = 0.46)	0.002
PAB responder rate (% ≥ 20%↑)	—	46.8%	*P* < 0.001	—	9.7%	*P* = 0.63	<0.001
BMI responder rate (%≥1 kg/m^2^↑)	—	39.2%	*P* < 0.001	—	8.3%	*P* = 0.58	<0.001

Δ represents mean change from baseline to follow-up within each group. Within-group comparisons were analyzed using paired *t*-test or Wilcoxon signed-rank test as appropriate. Between-group differences (ΔIntervention–ΔControl) were tested with independent *t*-test or Mann–Whitney U test. BMI, body mass index; PAB, prealbumin; NRS-2002, Nutritional Risk Screening 2002.

Following the intervention, significant within-group improvements were observed across all key nutritional indicators in the intervention cohort (all *p* < 0.01), including increases in body weight, BMI, albumin, prealbumin, and hemoglobin (Table 2). In contrast, changes in these parameters were minimal and non-significant in the control group (all *p* > 0.05). Direct between-group comparisons confirmed that the magnitude of improvement was consistently and significantly greater in the intervention group for all measures (e.g., between-group Δ in albumin: +2.5 g/L, *p* < 0.001) (Table 2).

A clear dose-response relationship was evident, as patients at higher baseline nutritional risk experienced the most substantial gains. For instance, the high-risk subgroup achieved greater increases in albumin and BMI compared to the low-risk subgroup (interaction *p* = 0.02). This risk-dependent benefit was further substantiated by a Linear Mixed-Effects Model, which, after multivariable adjustment, identified nutritional risk stratification management as an independent factor associated with improved albumin levels at follow-up (β = 2.34, 95% CI: 1.20–3.47, *p* < 0.001), with similar positive associations for prealbumin and BMI.

In this cohort, systematic nutritional risk stratification management was associated with improved nutritional status in IBD patients, with more pronounced improvements observed in high-risk populations. These results indicate that the stratified management model not only restores protein synthesis and body composition reserves but may also establish a foundation for subsequent disease control and quality of life improvement by ameliorating the nutrition-inflammation balance.

### 3.3 Changes in inflammation and disease activity

The intervention was associated with significant reductions in both systemic and intestinal inflammation markers. At follow-up, patients in the intervention group achieved markedly greater decreases in median CRP and fecal calprotectin (FCP) levels compared to the control group (between-group Δ difference: −4.7 mg/L for CRP and −92 μg/g for FCP, both *p* < 0.001; Table 3). Consequently, a significantly higher proportion of intervention patients attained an FCP level < 150 μg/g (41.8% vs. 22.8%, *p* < 0.001), indicating better-controlled mucosal inflammation.

**TABLE 3:** Changes in inflammatory markers and disease activity within and between groups.

Variable	Intervention baseline	Intervention follow-up	Δ (within group, *p*)	Control baseline	Control follow-up	Δ (within group, *p*)	Between-group Δ (*p*)
C-reactive protein (CRP, mg/L, median [IQR])	15.2 (8.1–25.6)	9.6 (4.2–17.8)	−5.6 (*p* < 0.001)	14.8 (7.6–24.3)	13.9 (7.3–23.9)	−0.9 (*p* = 0.18)	<0.001
Fecal calprotectin (FCP, μg/g, median [IQR])	298 (176–480)	184 (98–321)	−114 (*p* < 0.001)	290 (170–462)	268 (160–440)	−22 (*p* = 0.09)	<0.001
FCP < 150 μg/g (%)	19.6%	41.8%	*P* < 0.001	20.1%	22.8%	*P* = 0.32	<0.001
Harvey–Bradshaw index (HBI, mean ± SD)	6.9 ± 3.2	4.1 ± 2.7	−2.8 (*p* < 0.001)	6.7 ± 3.1	6.1 ± 3.0	−0.6 (*p* = 0.07)	<0.001
Mayo score (mean ± SD)	5.8 ± 2.3	3.9 ± 2.0	−1.9 (*p* < 0.001)	5.6 ± 2.2	5.2 ± 2.1	0.4 (p = 0.10)	<0.001
HBI: time × Group interaction (LMM)	—	—	*P* < 0.001	—	—	—	—
Mayo: time × Group interaction (LMM)	—	—	*P* < 0.001	—	—	—	—
Alb→CRP (β, 95% CI)	—	—	β = −1.10 (−1.60 to −0.70), *p* < 0.001	—	—	—	—
PAB→FCP (β, 95% CI)	—	—	β = −28 (−45 to −11), *p* = 0.003	—	—	—	—
Alb–CRP correlation (*r*)	—	—	*r* = −0.42, *p* < 0.001	—	—	—	—
Subgroup interaction (high vs. low nutritional risk)	—	—	*P* = 0.03	—	—	—	—

Alb, albumin; PAB, prealbumin; CRP, C-reactive protein; FCP, fecal calprotectin; HBI, Harvey–Bradshaw index.

These biochemical improvements were paralleled by clinically relevant reductions in disease activity scores. For both Crohn’s disease (assessed by HBI) and ulcerative colitis (assessed by Mayo score), the magnitude of decrease was significantly greater in the intervention group (between-group Δ: −2.2 points for HBI, −1.5 points for Mayo, both *p* < 0.001). A Linear Mixed-Effects Model confirmed a significant time-by-group interaction (*p* < 0.001), suggesting that the clinical benefit accrued over the follow-up period.

Supporting a direct link between nutritional and inflammatory outcomes, regression analysis revealed that improvements in serum Alb and PAB were independently associated with reductions in CRP (β = −1.10 mg/L per 1 g/L Alb increase) and FCP (β = −28 μg/g per 0.04 g/L PAB increase), respectively (both *p* < 0.01). A moderate negative correlation was also observed between Alb and CRP (*r* = −0.42, *p* < 0.001).

The anti-inflammatory effect appeared most pronounced in patients at moderate-to-high nutritional risk at baseline (interaction *p* = 0.03), while consistent across disease types and medication classes. The robustness of the primary findings was confirmed through sensitivity analyses, including multiple imputation and propensity score weighting (all *p* < 0.01).

Overall, systematic nutritional risk stratification management was associated with significant reductions in objective inflammatory markers and with sustained improvements in disease activity, with the strength and direction of both findings mutually reinforcing (see Table 3).

### 3.4 Disease relapse rate and hospitalization outcomes

**FIGURE 2:**
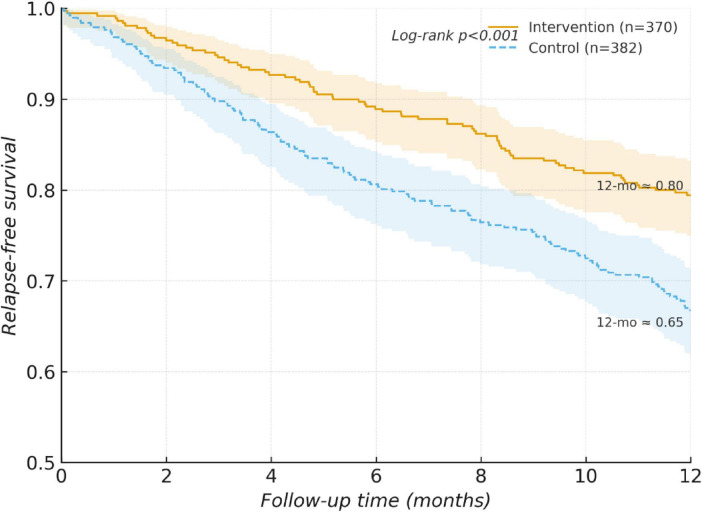
Relapse-free survival curves for the intervention and control groups. The median follow-up time was 9.8 months. The relapse-free survival rate was significantly higher in the intervention group (*n* = 370) compared to the control group (*n* = 382) (Log-rank *p* < 0.001). At 12 months, the relapse-free survival rate was approximately 0.80 in the intervention group and 0.65 in the control group, suggesting that exposure to systematic nutritional risk stratification management was associated with delayed disease relapse.

The median follow-up time was 9.8 months. The overall relapse rate was 27.7%, with a significantly lower proportion observed in the intervention group (20.5%) compared to the control group (34.8%) (*p* < 0.001). This difference translated to a significantly higher relapse-free survival probability for the intervention group throughout follow-up (log-rank *p* < 0.001; Figure 2).

**FIGURE 3:**
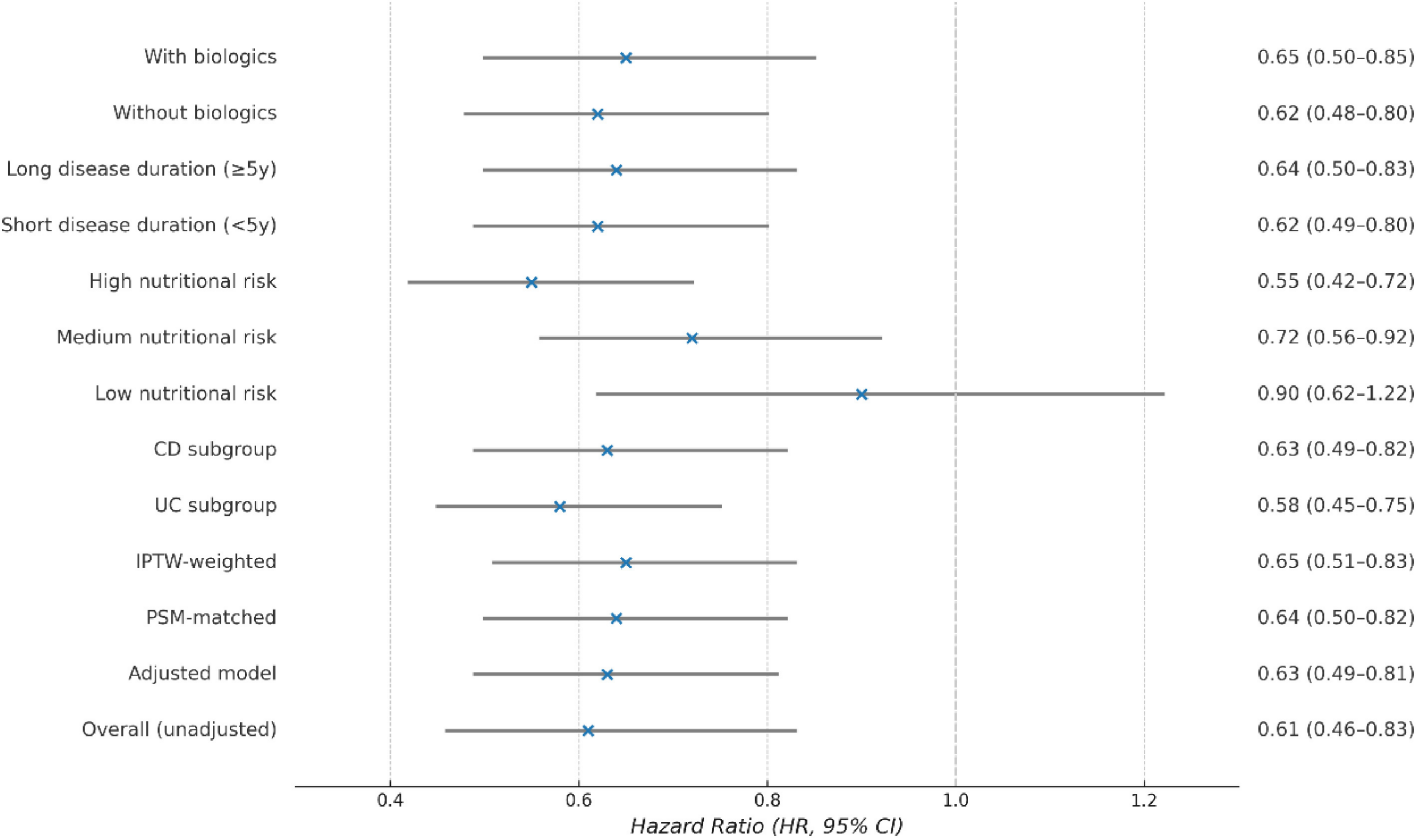
Effect of nutritional intervention on disease relapse risk across subgroups and models. Multivariable cox regression analysis demonstrated that systematic nutritional management was consistently associated with a lower risk of relapse across multiple subgroups, although these findings remain observational. The similarity of estimates across subgroups and models supports the robustness of this association but does not prove causality.

Cox regression analyses consistently demonstrated a significant reduction in relapse risk associated with the nutritional management pathway. In the primary adjusted model, the HR was 0.61 (95% CI: 0.46–0.83, *p* = 0.001). The robustness of this association was confirmed through sensitivity analyses employing propensity score matching (PSM-adjusted HR = 0.63) and IPTW, yielding similar estimates (Figure 3).

In a complementary time-dependent Cox model, in which entry into the dietitian-led nutritional pathway was treated as a time-varying exposure, the hazard ratio for relapse associated with nutritional management was very similar to that of the primary fixed-exposure analysis (Supplementary Table 1), suggesting that immortal time did not materially account for the observed association. In sensitivity analyses restricting relapse to objectively defined events (FCP > 250 μg/g plus IBD-related hospitalization or endoscopic/imaging worsening), the direction and magnitude of the association between nutritional risk stratification management and relapse remained similar to the primary analysis (Supplementary Table 8).

Subgroup analyses confirmed the consistency and specificity of the intervention’s benefit (Table 4). The association with reduced relapse risk was observed in both UC (HR = 0.57) and CD (HR = 0.65) patients. A significant interaction was noted by baseline nutritional risk level, with high-risk patients deriving the greatest benefit (interaction *p* = 0.02), supporting a “risk-dependent” effect.

**TABLE 4:** Relapse and hospitalization outcomes.

Parameter	Intervention (*n* = 370)	Control (*n* = 382)	Statistic/HR/ OR (95% CI)	*P*-value	Interpretation
Relapse rate (%)	20.5	34.8	χ^2^ = 15.21	<0.001	Significantly lower relapse in intervention group
Relapse-free survival (12 months)	79.5	65.2	Log-rank	<0.001	Curve separation maintained across follow-up (Figure 2)
Cox model (unadjusted)	—	—	HR = 0.55 (0.41–0.74)	<0.001	Associated with approximately 45% lower relapse hazard
Cox model (adjusted)	—	—	HR = 0.61 (0.46–0.83)	0.001	Association remained after multivariable adjustment
PSM model	—	—	HR = 0.63 (0.47–0.85)	0.002	Association remained after propensity score matching
UC subgroup	—	—	HR = 0.57 (0.42–0.78)	0.001	Greater benefit in UC patients
CD subgroup	—	—	HR = 0.65 (0.48–0.88)	0.008	Similar trend in CD
High nutritional risk subgroup	—	—	HR = 0.56 (0.39–0.81)	0.002	Risk-dependent benefit
Low nutritional risk subgroup	—	—	HR = 0.82 (0.60–1.12)	0.21	Not significant
IBD-related hospitalization rate (%)	12.7	20.9	χ^2^ = 6.79	0.009	Lower hospitalization risk
Median hospital stay (days)	6 (IQR 4–10)	8 (IQR 5–13)	Z = 2.44	0.015	Shorter stay in intervention group
Logistic regression (hospitalization)	—	—	OR = 0.58 (0.38–0.87)	0.008	Independent protective effect

Regarding healthcare utilization, the intervention was associated with significantly lower IBD-related hospitalization rates (12.7% vs. 20.9%, *p* = 0.009) and a shorter median hospital stay (6 vs. 8 days, *p* = 0.015). Logistic regression confirmed the pathway was independently associated with a reduced odds of hospitalization (OR = 0.58, 95% CI: 0.38–0.87, *p* = 0.008).

In this observational study, exposure to standardized nutritional risk stratification management was associated with a lower risk of relapse, lower hospitalization rates, and shorter hospital stays in IBD patients. The persistent association observed across multiple models suggests that this pathway may have a potential role in delaying recurrence of disease activity, but causal inferences cannot be confirmed from these data.

### 3.5 Changes in quality of life

**FIGURE 4:**
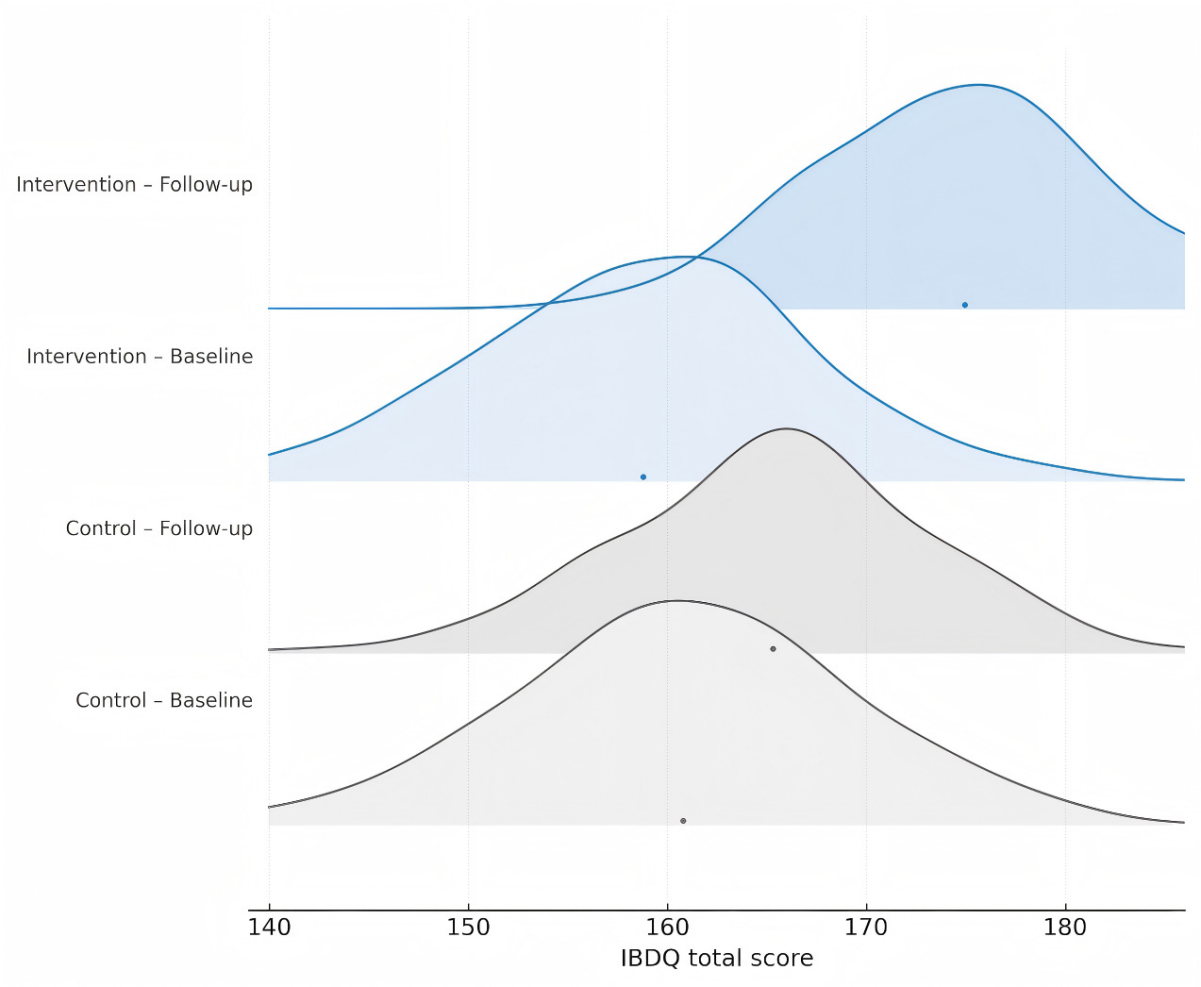
Distribution of changes in total IBDQ scores during follow-up for the intervention and control groups. Patients in the intervention group exhibited a significant increase in the total Inflammatory Bowel Disease Questionnaire (IBDQ) score during the follow-up period, whereas changes in the control group were minimal. The distribution curve for the intervention group shifted notably to the right, reflecting a significant improvement in quality of life. In contrast, the distribution curves for the control group largely overlapped, indicating that routine follow-up did not confer substantial improvement.

Systematic nutritional management was associated with a significant and clinically meaningful improvement in health-related quality of life. Among patients who completed the IBDQ questionnaire (*n* = 458), the total score increased substantially in the intervention group (Δ = +13.5, *p* < 0.001), exceeding the recognized threshold for a clinically significant change (>10 points), while the change in the control group was minimal (*p* = 0.41). The improvement trajectories diverged significantly over time (time-by-group interaction *p* < 0.001; Figure 4).

**FIGURE 5:**
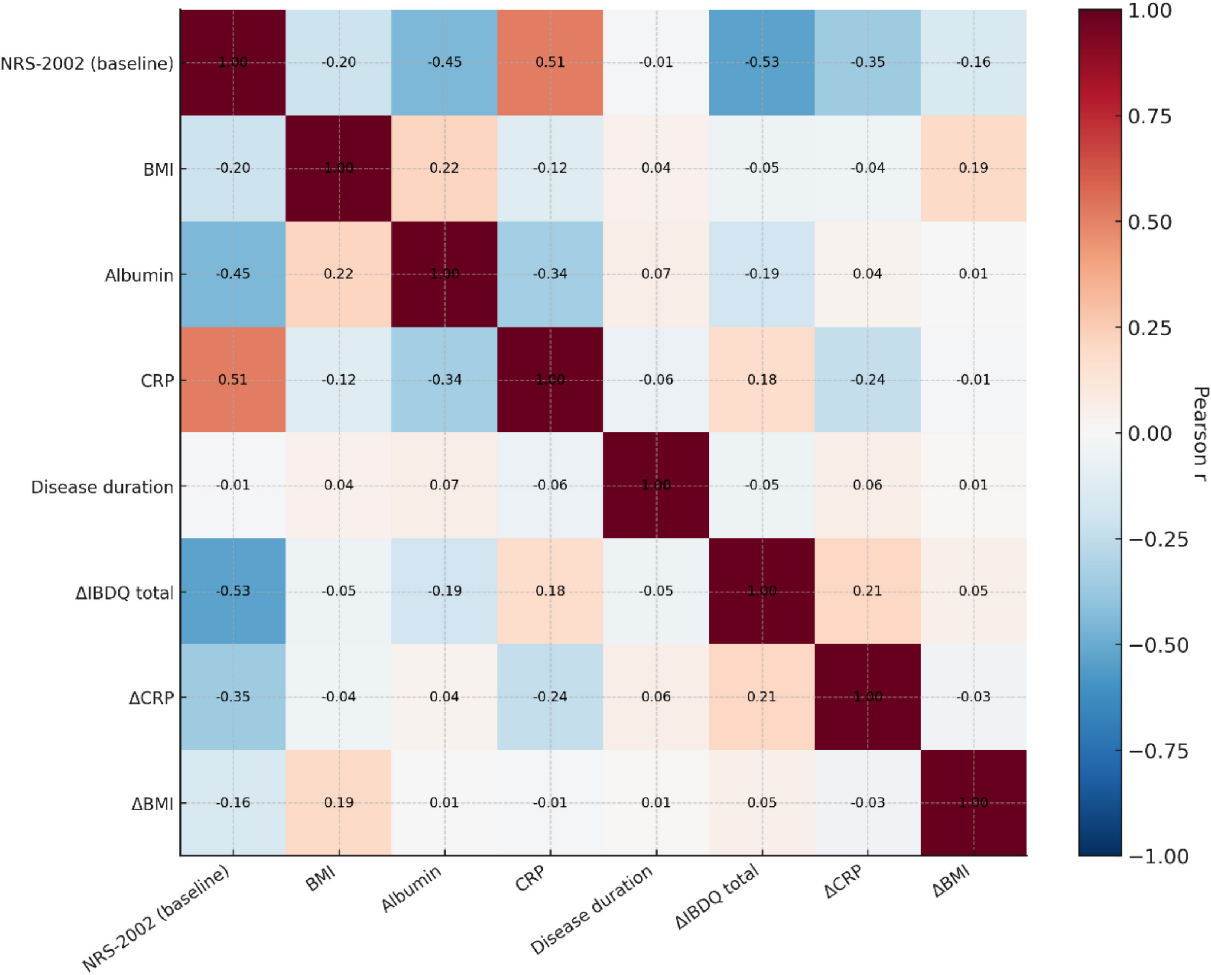
Correlation between Nutritional/Inflammatory Markers and Improvement in Quality of Life (ΔIBDQ). Pearson correlation coefficients between baseline nutritional status, inflammatory levels, and the magnitude of improvement in IBDQ scores (ΔIBDQ) are presented. The baseline NRS-2002 score showed a significant negative correlation with ΔIBDQ (*r* = −0.53, *p* < 0.001). CRP was positively correlated with ΔIBDQ, while albumin was negatively correlated with ΔIBDQ. This suggests that patients with higher baseline nutritional risk and more active inflammation derived greater benefit from the systematic intervention.

Correlation and regression analyses indicated that the magnitude of quality of life gain (ΔIBDQ) was significantly greater for patients with a higher baseline nutritional risk, as indicated by a negative correlation with the NRS-2002 score (*r* = −0.50, *p* < 0.001) and confirmed by linear regression (β = −2.1, *p* < 0.001; Table 5). This reinforces the “risk-dependent” benefit pattern observed for other outcomes (Figure 5).

**TABLE 5:** Correlation/regression analyses with baseline nutritional and inflammatory indicators.

Variable	Association with Δ IBDQ	R/R^2^	*P*
NRS-2002 (baseline)	β = −2.1(95% CI: −2.8 to −1.5)	R^2^ = 0.25	<0.001
NRS-2002 vs. ΔIBDQ	*r* = −0.50	—	<0.001
CRP (baseline) vs. ΔIBDQ	*r* = 0.25	—	<0.001
Albumin (baseline) vs. ΔIBDQ	*r* = −0.25	—	<0.001

**FIGURE 6:**
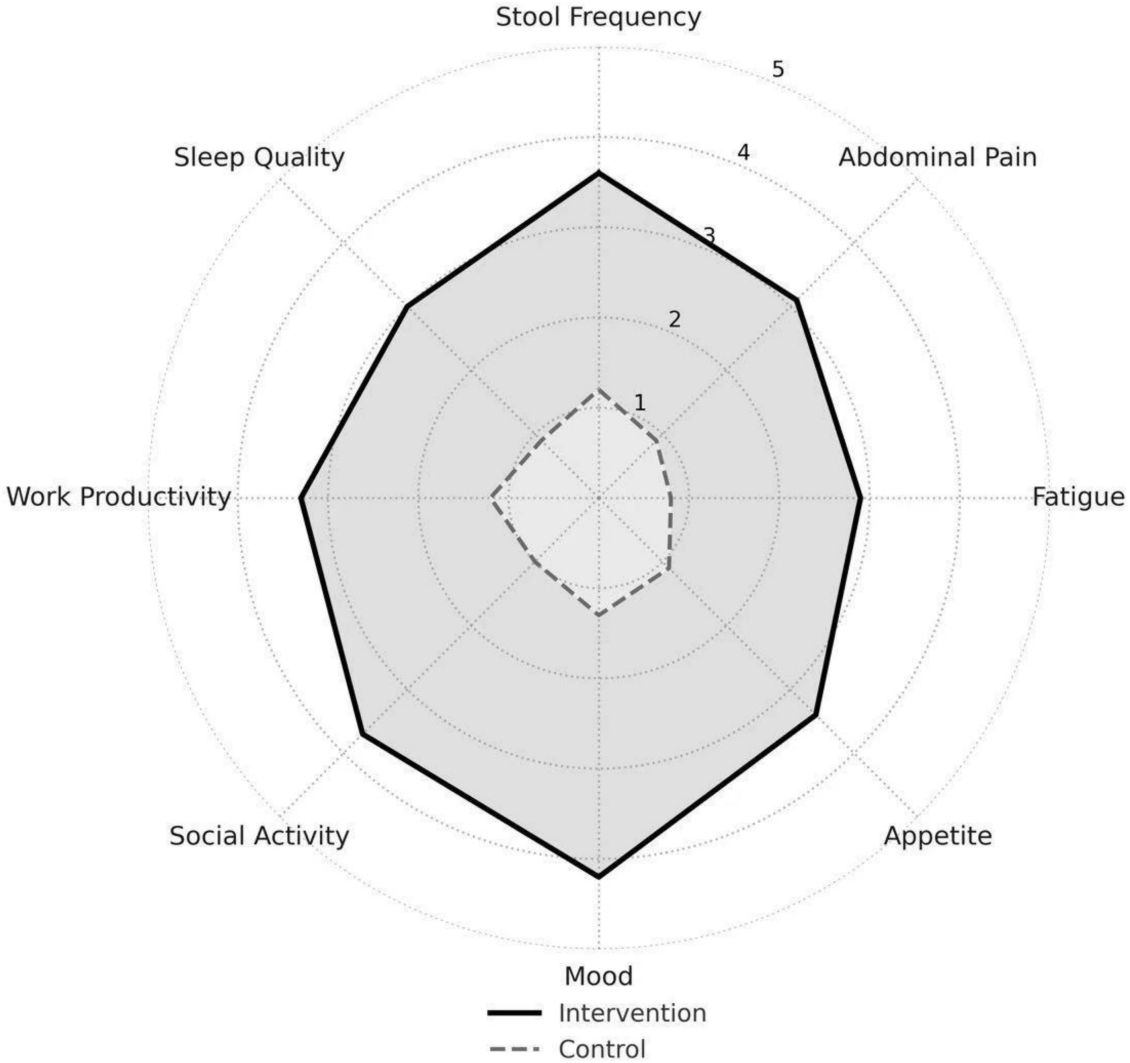
Inflammatory Bowel Disease Questionnaire (IBDQ) subdomain scores for the intervention and control groups. Patients in the intervention group showed varying degrees of improvement across all eight quality of life subdomains (including emotional function, bowel symptoms, social activity, work productivity, fatigue, sleep quality, appetite, and defecation frequency), with the most notable improvements observed in emotional function, bowel symptoms, social activity, and work productivity. Changes in the control group were limited. The results indicate that systematic nutritional management facilitates multidimensional and balanced improvement in quality of life.

Improvements were observed across all IBDQ subdomains, with the most notable gains in emotional function, bowel symptoms, and social activity (Figure 6). This multi-dimensional enhancement suggests that the benefits of structured nutritional care extend beyond physical health to encompass key aspects of psychosocial well-being and daily functioning.

### 3.6 Subgroup and sensitivity analyses

To evaluate the stability of the nutritional risk stratification management effect, further analyses were conducted across different clinical subgroups (see Table 6). The results showed that the direction of the intervention’s effect on relapse risk was consistent across subgroups defined by disease type (UC vs. CD), baseline disease activity (mild-moderate vs. severe), nutritional risk level (low, moderate, high), and treatment regimen (use of biologics or immunomodulators vs. not). None of the interaction terms reached statistical significance (all interaction *p* > 0.1). This indicates that the relapse risk reduction effect of systematic nutritional management is generalizable and not significantly modified by disease subtype or treatment strategy differences. Notably, the intervention effect was most pronounced in moderate-to-high nutritional risk populations and those with higher baseline disease activity (HR = 0.56 and 0.51, respectively), reinforcing the “risk-dependent” nature of the benefit from stratified management. Patients with poorer baseline nutritional status or higher inflammatory burden experienced greater reductions in relapse risk from nutritional intervention. This finding is consistent with the previous analyses of quality of life and inflammatory markers, and is compatible with a potential role of improved nutritional status in disease control.

**TABLE 6:** Subgroup and sensitivity analyses.

Analysis type	Subgroup/adjustment	Comparison	HR (95% CI)	*P*-value
Subgroup analysis	Disease type (UC vs. CD)	Intervention vs. control	0.60 (0.45–0.82)	0.002
Subgroup analysis	Baseline activity (high vs. low)	Intervention vs. control	0.51 (0.36–0.73)	0.001
Subgroup analysis	Nutrition risk (low/medium/high)	Intervention vs. control	0.56 (0.40–0.78)	0.001
Subgroup analysis	Treatment regimen (±biologics)	Intervention vs. control	0.62 (0.44–0.86)	0.005
Sensitivity analysis	Multiple imputation (*m* = 20)	Intervention vs. control	0.61 (0.46–0.83)	0.001
Sensitivity analysis	IPTW weighted model	Intervention vs. control	0.59 (0.44–0.81)	0.001
Sensitivity analysis	E-value analysis	—	E-value = 2.01	—

To verify the robustness of the results, Multiple Imputation (*m* = 20) and IPTW analyses were performed. The results of the Cox proportional hazards model after multiple imputation were consistent with the complete case analysis (HR≈0.61, 95% CI: 0.46–0.83), suggesting that missing data did not significantly affect the primary conclusions. The IPTW analysis also remained robust (weighted HR = 0.59, 95% CI: 0.44–0.81, *p* = 0.001), indicating that nutritional stratification management remained independently associated with reduced relapse risk after weighting adjustment. Furthermore, an E-value analysis yielded a result of 2.01, suggesting that an unmeasured confounder would need to be associated with both the exposure and outcome by a risk ratio of approximately 2.01 each, above and beyond the measured covariates, to fully explain away the observed association. However, an E-value of this magnitude is modest and compatible with the presence of plausible unmeasured confounders in routine IBD care; therefore, the analysis should be interpreted as quantifying the robustness of the association to measured covariates rather than ruling out residual confounding. Together, the subgroup and sensitivity analyses suggest that nutritional risk stratification management is consistently associated with lower relapse risk across different patient subgroups, supporting a potential role for this pathway as a component of long-term IBD management while underscoring that causal inferences cannot be definitively drawn from these data.

## 4 Discussion

Malnutrition is a highly prevalent and clinically significant comorbidity in IBD, with multifactorial origins encompassing reduced oral intake, nutrient malabsorption, and a chronic inflammatory state that elevates metabolic demands. It affects a substantial proportion of patients, with estimates ranging from 20% to 80% across different cohorts (9). Critically, malnutrition is not merely a secondary consequence but an independent driver of adverse outcomes, contributing to an increased risk of disease relapse, suboptimal response to therapy, and a diminished quality of life (26). Against this established backdrop, our study was designed to evaluate the real-world effectiveness of a systematic nutritional risk stratification management pathway in a cohort of IBD patients.

Our primary findings indicate that this structured, dietitian-led pathway was associated with significant and multi-faceted benefits relative to conventional care. The intervention group demonstrated superior recovery of nutritional status, evidenced by more pronounced gains in body weight and key serological markers like albumin. This outcome aligns with and extends evidence from previous controlled trials, such as those demonstrating the nutritional efficacy of the Mediterranean diet in Crohn’s disease (27) or the value of preoperative nutritional optimization (28, 29). Our results suggest that a pragmatic, risk-stratified program implemented in a routine clinical setting can successfully translate these principles into tangible patient benefit, reinforcing the call for personalized, dietitian-guided nutritional strategies to improve adherence and outcomes (30).

In parallel, we observed a consistent reduction in objective markers of systemic and intestinal inflammation, namely CRP and fecal calprotectin, alongside improvements in standardized disease activity indices. This supports the biologically plausible concept that sustained nutritional repletion and modulation can mitigate underlying inflammatory processes in IBD. The observed effect aligns with clinical trials of structured nutritional regimens, such as the Crohn’s Disease Exclusion Diet, which have demonstrated efficacy in reducing gut inflammation (22), and with mechanistic studies indicating that nutritional therapies may promote mucosal healing (31). While short-term, isolated dietary modifications may not invariably produce significant shifts in systemic markers like CRP (32), our study—featuring a longer-term, multimodal intervention—suggests that a more comprehensive and sustained nutritional approach can contribute to inflammatory downregulation.

A particularly compelling finding was the association of the nutritional management pathway with a reduced incidence of disease relapse and fewer IBD-related hospitalizations. This directly addresses a major clinical concern, as malnutrition is a well-recognized risk factor for disease recurrence (33). By systematically improving a patient’s nutritional substrate, the intervention may help disrupt the vicious cycle wherein inflammation drives catabolism and malnutrition, which in turn may perpetuate immune dysregulation. Our results thus provide supportive real-world evidence for the potential role of nutritional strategies as an adjunct to pharmacotherapy in maintaining remission, a notion previously suggested in studies of maintenance-phase enteral nutrition (34).

Finally, and of direct importance to patients, the intervention group achieved significantly greater improvements in HRQoL, meeting thresholds for clinically meaningful change. Malnutrition profoundly impacts HRQoL through fatigue, functional impairment, and psychosocial strain (35). The individualized nutritional support and counseling provided in our pathway likely contributed to symptom alleviation and restored physical capacity, thereby enhancing daily functioning and social participation. This observation corroborates international reports on the positive impact of dietary management on overall well-being in IBD (36) and underscores to clinicians that nutritional care is integral to achieving holistic treatment success, complementing the control of objective disease markers.

This study has several limitations. First, as a retrospective study, its non-randomized design indicates that the observed associations should not be interpreted as causal. Exposure to the nutritional pathway was determined by clinical practice, and despite multivariable adjustment, propensity score methods, and E-value analysis, unmeasured confounding (e.g., unrecorded socio-economic factors, nuanced disease severity, or health-seeking behaviors) likely persists. Second, selection bias and limited generalizability must be acknowledged, as only 39.8% (752/1,892) of the managed patient population was included. A substantial number of patients were excluded due to missing nutritional screening (*n* = 604), insufficient follow-up (<3 months, *n* = 386), or missing key data (*n* = 150), patterns detailed in Supplementary Tables 5, 6; these individuals may differ systematically from the analytic cohort. Third, the intervention was a heterogeneous, pragmatic care package (with specific components and durations summarized in Supplementary Table 2) rather than a standardized protocol, and potential exposure misclassification in the control group (e.g., self-initiated supplement use, addressed in per-protocol sensitivity analyses referenced in Supplementary Table 3) likely biases effect estimates toward the null. Fourth, while design features (e.g., a strict 14-day exposure window) and a time-dependent Cox analysis were employed to address immortal time bias, residual time-related bias cannot be entirely excluded. Fifth, the need to harmonize two screening tools (NRS-2002 and MUST) into a single risk variable may introduce some misclassification of baseline nutritional status. Sixth, part of the observed benefit may be attributable to closer monitoring and more frequent contact embedded within the pathway, rather than nutritional improvement alone. Finally, the single-center setting and median follow-up of approximately 10 months limit the external validity and assessment of the long-term durability of the associations. Taken together, these limitations underscore that our findings represent evidence of association and highlight the need for prospective, randomized trials to establish causality.

## 5 Conclusion

Based on real-world clinical data from a single-center retrospective cohort, this study found that exposure to systematic nutritional risk stratification management was associated with better nutritional status, lower inflammatory markers and disease activity, reduced relapse and hospitalization rates, and improved quality of life in patients with inflammatory bowel disease. These findings support the hypothesis that integrating structured nutritional risk screening, individualized nutrition support, and dynamic reassessment into routine IBD care may contribute to improved clinical outcomes. However, because of the observational design, potential selection and information bias, exposure misclassification, and residual confounding, the observed associations should not be interpreted as definitive causal effects. Future prospective multicenter randomized studies and implementation research are needed to confirm these findings, clarify underlying mechanisms, and determine how best to incorporate nutritional risk stratification management into evidence-based, patient-centered IBD care pathways.

## Data Availability

The raw data supporting the conclusions of this article will be made available by the authors, without undue reservation.
