# Supplementary material for: Nutritional risk stratification management is associated with reduced disease relapse and improved quality of life in IBD patients: a retrospective study
Source: Front Nutr. 2026 Jan 12;12:1729247. doi: 10.3389/fnut.2025.1729247 (PMC12832400; doi:10.3389/fnut.2025.1729247)
Supplement: Supplementary file 1 [file Data_Sheet_1.docx]

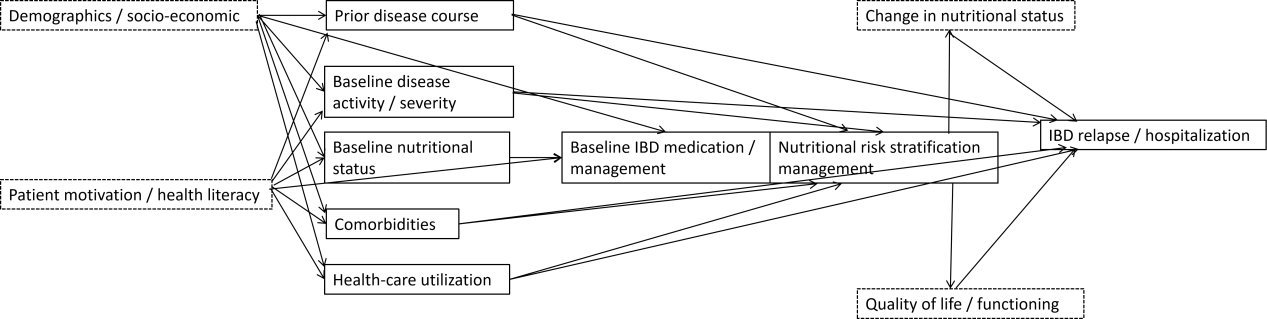


**Supplementary Figure 1. Directed acyclic graph (DAG) illustrating the assumed causal structure between nutritional risk stratification management and IBD relapse/hospitalization.**
The exposure is “nutritional risk stratification management” (dietitian-led pathway combining dietary education, ONS, EN/PN, and scheduled reassessment), and the outcome is IBD relapse or hospitalization. Measured baseline covariates (solid boxes) include prior disease course, baseline disease activity/severity, baseline nutritional status, comorbidities, baseline IBD medication/disease management, and health-care utilization, which may influence both entry into the nutritional pathway and subsequent relapse risk. Demographic and socio-economic characteristics, patient motivation and health literacy, changes in nutritional status, and quality of life/functioning are represented as unmeasured or partially measured factors (dashed boxes). The DAG was used to guide selection of confounders for multivariable Cox models and propensity score analyses and to distinguish potential mediators (change in nutritional status and quality of life) from baseline confounders.


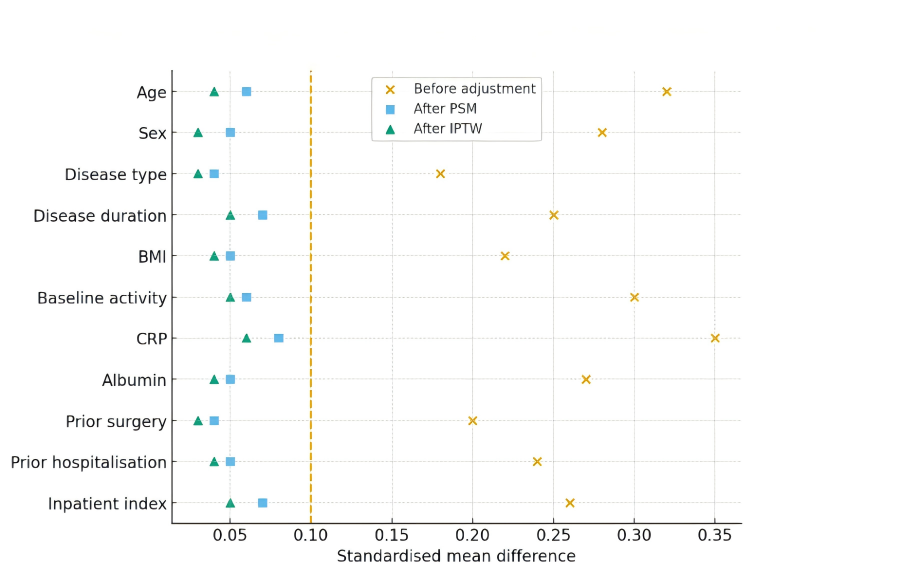


Supplementary Figure S2. Covariate balance before and after propensity score matching (PSM) and inverse probability of treatment weighting (IPTW).
Standardised mean differences (SMDs) for each covariate are shown for the original sample (before adjustment), the matched sample (after PSM), and the weighted pseudo-population (after IPTW). The vertical dashed line at SMD = 0.10 indicates the conventional threshold for acceptable balance.

Supplementary Table S1. Sensitivity analysis for the association between nutritional risk stratification management and IBD relapse using fixed-exposure and time-dependent Cox models

| **Model** | **Exposure definition** | **Covariate adjustmenta** | **HR for relapse (95% CI)** | **p value** |
| --- | --- | --- | --- | --- |
| Model 1 | Fixed baseline exposure (intervention vs. control)b | None (unadjusted) | 0.68 (0.53–0.88) | 0.003 |
| Model 2 | Fixed baseline exposure (intervention vs. control)b | Age, sex, disease type, disease duration, baseline disease activity, baseline nutritional status, CRP, albumin, prior surgery, hospitalization in previous 12 months, care setting | 0.62 (0.47–0.82) | 0.001 |
| Model 3 | Time-dependent exposure (0 = conventional care, 1 = dietitian-led pathway from date of entry)c | Same as Model 2 | 0.64 (0.49–0.84) | 0.002 |

Note:

^a^ Multivariable models were adjusted for pre-specified baseline confounders identified from prior literature and an a priori directed acyclic graph (DAG), including age, sex, disease type (UC vs. CD), disease duration, baseline clinical disease activity, baseline nutritional status (NRS-2002/MUST, BMI, albumin), C-reactive protein (CRP), prior IBD-related surgery, hospitalization in the previous 12 months, and care setting at index (inpatient vs. outpatient).

^b^ In Models 1–2, exposure status (nutritional risk stratification management vs. conventional care) was determined at time zero based on whether the patient entered the dietitian-led nutritional pathway within 14 days of the index date. Patients who crossed over to the structured pathway after this window were censored at the time of crossover.

^c^ In Model 3, entry into the dietitian-led nutritional pathway was modeled as a time-dependent covariate that changed from 0 (conventional care only) to 1 (structured pathway) on the date of first pathway entry; patients who never entered the pathway remained at 0 throughout follow-up.

Supplementary Table S2. Intervention components, duration and adherence in the dietitian-led nutritional pathway

| **Intervention Component / Metric** | **Value** |
| --- | --- |
| **Intervention composition, n (%):** |  |
| – Dietary education only | 104 (28.1%) |
| – Education + ONS only | 118 (31.9%) |
| – Education + EN (± ONS), no PN | 98 (26.5%) |
| – Any PN (± EN/ONS) | 50 (13.5%) |
| **Escalation observed, n (%):** |  |
| – Education → ONS | 122 (33.0%) |
| – ONS → EN | 65 (17.6%) |
| – EN → PN | 21 (5.7%) |
| **Duration of each modality, median weeks (IQR):** |  |
| – ONS | 8.0 (5.0–13.0) |
| – EN | 5.0 (3.0–9.0) |
| – PN | 3.0 (2.0–6.0) |
| **Follow-up intensity (12 months), median (IQR):** |  |
| – In-person dietitian visits | 4 (3–6) |
| – Structured telephone contacts | 3 (2–5) |
| **Adherence metrics:** |  |
| – Achieved energy intake (% of prescribed) | 89% (76–95%) |
| – Achieved protein intake (% of prescribed) | 87% (74–93%) |
| – Good contact adherencea, n (%) | 296 (80.0%) |
| – Good ONS/EN adherenceb, n (%) | 215 (58.1%) |

Note: a Defined as attending ≥80% of planned dietitian contacts.
b Defined as consuming ≥75% of prescribed ONS/EN on ≥80% of follow-up days.

Supplementary Table S3. Per-protocol and contamination-sensitive analyses of the association between nutritional risk stratification management and relapse

| **Analysis population** | **Exposure definition / handling of contamination** | **HR for relapse (95% CI)** | **p value** |
| --- | --- | --- | --- |
| **Main analysis** (as-treated) | Control group includes self-initiated ONS use | 0.62 (0.47–0.82) | 0.001 |
| **Per-protocol 1** | Excludes control patients who self-initiated ONS | 0.60 (0.45–0.80) | 0.001 |
| **Per-protocol 2** | Reclassifies self-initiated ONS users as "partially exposed" | 0.63 (0.48–0.83) | 0.001 |

Note: Self-initiated ONS refers to oral nutritional supplement use not linked to dietitian prescription or pathway entry, reported in medical records or pharmacy logs.

Supplementary Table S4 Sensitivity analysis restricted to NRS-2002 patients (n = 540)

| **Model** | **Exposure** | **HR for relapse (95% CI)** | **p value** |
| --- | --- | --- | --- |
| 1 | Unadjusted | 0.69 (0.51–0.93) | 0.015 |
| 2 | Adjusted Cox | 0.63 (0.46–0.86) | 0.004 |
| 3 | IPTW-adjusted | 0.61 (0.45–0.84) | 0.003 |

Note: All models adjusted for key covariates as in main analysis.

Supplementary Table S5 Comparison of baseline characteristics between included and excluded patients

| **Characteristic** | **Included (n = 752)** | **Excluded (n = 1,140)** | **p value** |
| --- | --- | --- | --- |
| Age, years (mean ± SD) | 37.9 ± 13.1 | 39.5 ± 15.4 | 0.028 |
| Female sex, n (%) | 396 (52.7%) | 605 (53.1%) | 0.88 |
| Ulcerative colitis, n (%) | 408 (54.3%) | 636 (55.8%) | 0.54 |
| Crohn’s disease, n (%) | 344 (45.7%) | 504 (44.2%) | — |
| Disease duration, years (median, IQR) | 3.8 (1.2–7.5) | 4.2 (1.5–8.0) | 0.12 |
| BMI, kg/m² (mean ± SD) | 21.4 ± 3.7 | 22.1 ± 3.9 | 0.002 |
| Clinical remission, n (%) | 412 (54.8%) | 710 (62.3%) | 0.001 |
| Mild activity, n (%) | 174 (23.1%) | 215 (18.9%) |  |
| Moderate–severe activity, n (%) | 166 (22.1%) | 215 (18.8%) |  |
| CRP, mg/L (median, IQR) | 5.2 (2.1–12.8) | 3.7 (1.5–9.4) | 0.008 |
| Albumin, g/L (mean ± SD) | 38.2 ± 4.5 | 39.1 ± 4.2 | 0.003 |
| Prior IBD-related surgery, n (%) | 124 (16.5%) | 174 (15.3%) | 0.52 |
| Hospitalization in prior 12 mo, n (%) | 156 (20.7%) | 144 (12.6%) | <0.001 |
| Inpatient at index, n (%) | 274 (36.4%) | 315 (27.6%) | <0.001 |

Note: Values are presented as mean ± standard deviation, median (interquartile range), or number (percentage), as appropriate.

Supplementary Table S6 Comparison of baseline characteristics between patients with and without documented NRS-2002/MUST screening among the full IBD source population (n = 1,892)

| **Characteristic** | **With screening (n = 1,288)** | **Without screening (n = 604)** | **p value** |
| --- | --- | --- | --- |
| Age, years (mean ± SD) | 38.2 ± 13.9 | 40.1 ± 15.1 | 0.017 |
| Female sex, n (%) | 690 (53.6%) | 311 (51.5%) | 0.42 |
| Ulcerative colitis, n (%) | 716 (55.6%) | 328 (54.3%) | 0.61 |
| Crohn’s disease, n (%) | 572 (44.4%) | 276 (45.7%) | — |
| Disease duration, years (median, IQR) | 4.0 (1.4–7.8) | 4.3 (1.6–8.1) | 0.15 |
| BMI, kg/m² (mean ± SD) | 21.7 ± 3.8 | 22.3 ± 4.1 | 0.004 |
| Clinical remission, n (%) | 740 (57.5%) | 382 (63.3%) | 0.009 |
| Moderate–severe activity, n (%) | 262 (20.3%) | 102 (16.9%) |  |
| CRP, mg/L (median, IQR) | 5.0 (2.0–11.5) | 3.5 (1.4–8.7) | 0.002 |
| Albumin, g/L (mean ± SD) | 38.3 ± 4.6 | 39.0 ± 4.2 | 0.006 |
| Hospitalization in prior 12 mo, n (%) | 268 (20.8%) | 85 (14.1%) | <0.001 |
| Inpatient at index, n (%) | 456 (35.4%) | 133 (22.0%) | <0.001 |

Supplementary Table S7 Effective sample size and covariate balance after IPTW

| **Covariate** | **SMD before IPTW** | **SMD after IPTW** |
| --- | --- | --- |
| Age | 0.32 | 0.04 |
| Sex | 0.28 | 0.03 |
| Disease type (UC vs CD) | 0.18 | 0.03 |
| Disease duration | 0.25 | 0.05 |
| BMI | 0.22 | 0.04 |
| Baseline activity | 0.3 | 0.05 |
| CRP | 0.35 | 0.06 |
| Albumin | 0.27 | 0.04 |
| Prior surgery | 0.2 | 0.03 |
| Prior hospitalisation | 0.24 | 0.04 |
| Inpatient at index | 0.26 | 0.05 |

Effective sample size (post-IPTW): 718 (from original n = 752)

Supplementary Table S8 Sensitivity analysis using an objective relapse definition

| **Model** | **Relapse definition** | **Exposure specification** | **HR for relapse (95% CI)** | **p value** |
| --- | --- | --- | --- | --- |
| 1 | FCP >250 μg/g + IBD-related hospitalisation or endoscopic/imaging worsening | Fixed baseline exposure (intervention vs. control) | 0.70 (0.52–0.95) | 0.021 |
| 2 | Same as above | Multivariable-adjusted | 0.66 (0.49–0.90) | 0.009 |
| 3 | Same as above | Time-dependent exposure, adjusted | 0.67 (0.50–0.91) | 0.011 |

Note: Exposure defined as entry into the nutritional pathway within 14 days of baseline. Time-dependent analysis models pathway entry as a time-varying covariate.

Supplementary Table S9 Benjamini–Hochberg FDR-adjusted p-values for secondary outcomes

| **Outcome** | **Model** | **Raw p-value** | **FDR-adjusted p-value** |
| --- | --- | --- | --- |
| Relapse frequency | Negative binomial regression | 0.004 | 0.008 |
| IBD-related hospitalisation (yes/no) | Logistic regression | 0.011 | 0.016 |
| Number of hospitalisations | Negative binomial regression | 0.019 | 0.023 |
| Length of stay | Linear regression | 0.032 | 0.038 |
| IBDQ score improvement | LMM | 0.002 | 0.008 |
| Serum albumin change | LMM | 0.006 | 0.012 |
| CRP change | LMM | 0.015 | 0.021 |
| Nutritional risk improvement | Logistic regression | 0.027 | 0.035 |
